# Efficient CRISPR/Cas9-mediated genome modification of the glassy-winged sharpshooter *Homalodisca vitripennis* (Germar)

**DOI:** 10.1038/s41598-022-09990-4

**Published:** 2022-04-19

**Authors:** Inaiara de Souza Pacheco, Anna-Louise A. Doss, Beatriz G. Vindiola, Dylan J. Brown, Cassandra L. Ettinger, Jason E. Stajich, Richard A. Redak, Linda L. Walling, Peter W. Atkinson

**Affiliations:** 1 Department of Entomology, University of California, Riverside, CA 92521 USA; 2 Department of Microbiology & Plant Pathology, University of California, Riverside, CA 92521 USA; 3 Department of Botany & Plant Sciences, University of California, Riverside, CA 92521 USA; 4 Institute for Integrative Genome Biology, University of California, Riverside, CA 92521 USA

**Keywords:** Biotechnology, Genetics, Molecular biology

## Abstract

CRISPR/Cas9 technology enables the extension of genetic techniques into insect pests previously refractory to genetic analysis. We report the establishment of genetic analysis in the glassy-winged sharpshooter (GWSS), *Homalodisca vitripennis*, which is a significant leafhopper pest of agriculture in California. We use a novel and simple approach of embryo microinjection in situ on the host plant and obtain high frequency mutagenesis, in excess of 55%, of the *cinnabar* and *white* eye pigmentation loci. Through pair matings, we obtained 100% transmission of *w* and *cn* alleles to the G3 generation and also established that both genes are located on autosomes. Our analysis of wing phenotype revealed an unexpected discovery of the participation of pteridine pigments in wing and wing-vein coloration, indicating a role for these pigments beyond eye color. We used amplicon sequencing to examine the extent of off-target mutagenesis in adults arising from injected eggs, which was found to be negligible or non-existent. Our data show that GWSS can be easily developed as a genetic model system for the Hemiptera, enabling the study of traits that contribute to the success of invasive pests and vectors of plant pathogens. This will facilitate novel genetic control strategies.

## Introduction

CRISPR/Cas9 technology provides the means to direct mutagenesis to a specific site in a genome; its application to insect pests brings genetic control strategies to the forefront in insect species where genetic manipulations have either proven elusive or not been attempted. All that is required for direct mutagenesis is a high-quality draft genome, a means to introduce the Cas9 protein and single-guide RNAs (sgRNAs) to the germline, and the ability to perform genetic crosses. Here we describe deployment of this technology in *Homalodisca vitripennis* Germar (glassy-winged sharpshooter, GWSS). GWSS is a polyphagous hemipteran pest that, while invasive to California, is native to the south-eastern United States and north-eastern Mexico. It is a xylem-feeder with more than 100 host plants^1^ and is an important vector of *Xylella fastidiosa,* which causes Pierce’s Disease (PD) in grapes and other pathologies its host plants^1–3^. The costs of PD alone are considerable, as GWSS endangers the Californian grape and wine industry that generates $57.6 billion annually^4,5^. Control of GWSS has focused on quarantine restrictions across California, as well as the application of chemical insecticides. Resistance to pyrethroid and neonicotinoid insecticides has emerged indicating that the use of these broad-spectrum insecticides may be short lived^6,7^. New approaches for the control of this pest, and other hemipteran pests of agriculture, are urgently needed. These control strategies should provide specificity by targeting only the populations and species of concern and be economically and environmentally sustainable.

RNAi-based technology has been successfully deployed in more than 30 species across all suborders of the Hemiptera and has provided insight into mechanisms of insecticide resistance, immunity, reproduction, behavior, and metabolite biosynthesis^8^. Unfortunately, RNAi-based approaches cannot produce null mutations nor stable genetic lines and are prone to substantial variation in phenotypes based on the mode and timing of delivery of dsRNAs. In contrast, CRISPR/Cas9 genetic technologies accelerate the ability to generate and test genetic-based control technologies for pests. Extension of this technology into species in Diptera, Lepidoptera and Coleoptera was relatively rapid^9–11^. In contrast, CRISPR/Cas9-mediated knock-out mutagenesis in the Hemiptera was not reported until 2018. To date, this technology has been used in seven species within six families of the Hemiptera including: brown planthopper (*Nilaparvata lugens*), corn planthopper (*Peregrinus maidis*), milkweed bug (*Oncopeltus fasciatus)*, linden bug (*Pyrrhocoris apterus)*, neotropical stink bug (*Euschistus heros)*, whitefly (*Bemisia tabaci)*, and pea aphid (*Acyrthosiphon pisum)*^12–21^.

Here we report the establishment of an easy-to-deploy, high-frequency CRISPR/Cas9-mediated genetic analysis platform for GWSS. Stable lines with mutations in two eye-pigmentation genes, *cinnabar* (*cn*) and *white* (*w*), were established and maintained for three or more generations. Through reciprocal pair matings we showed that neither gene is sex linked. We discovered that *w* mutants reduced or eliminated eye color, as well as diminished two classes of red pteridines of the veins and interveinal spaces of the forewing. In contrast, *cn* mutations resulted in insects with brilliant red–orange eye color and normal pigmentation of forewings; in addition, the unique patterning of ommatidial cells that expressed pteridines was revealed. The ease and high-efficiencies of the CRISPR/Cas9 technologies in GWSS combined with availability of an improved GWSS genome project^22^ enables genetic approaches for the control of this pest to be designed and pursued with the potential of GWSS emerging as a model Hemipteran organism^23^.

## Results

### Microinjection of GWSS embryos on leaf discs results in high survival

**Figure 1:**
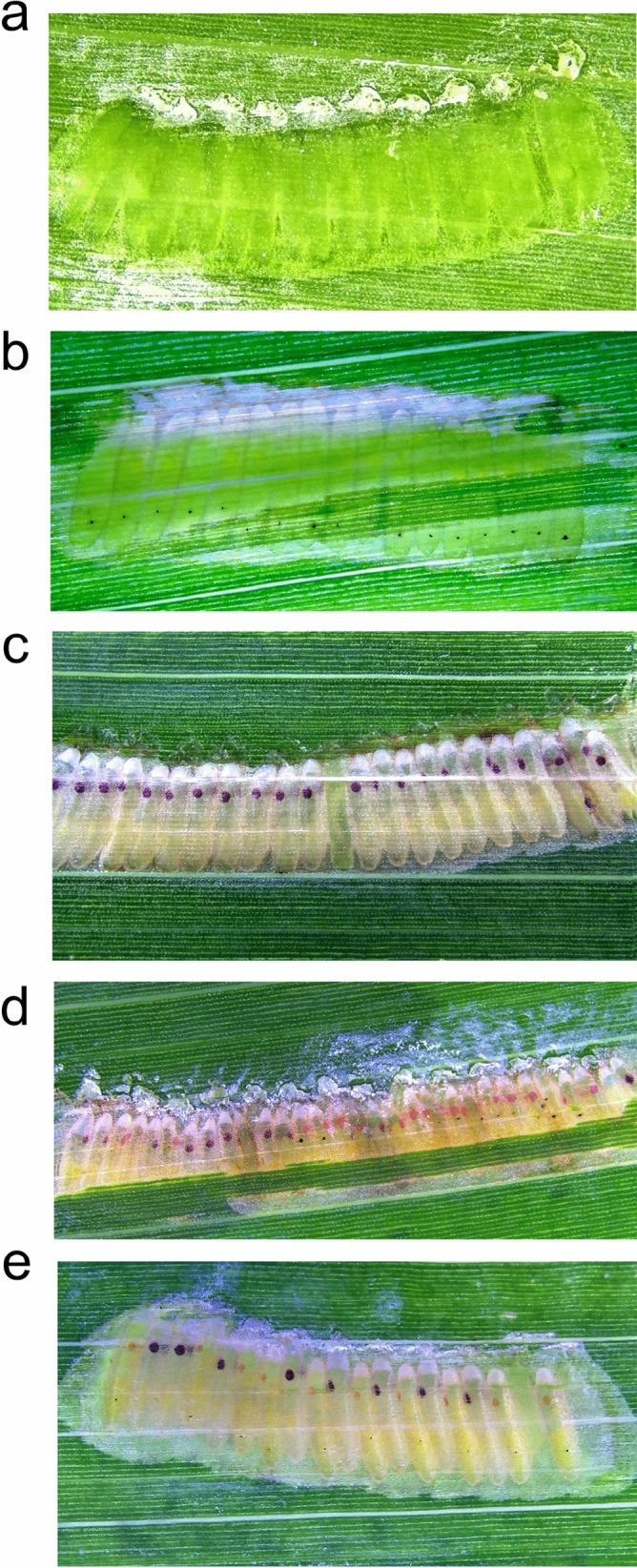
Phenotypes of GWSS egg masses pre- and post-njection. (**a**) Egg mass prior to injection (n = 20). The side-by-side deposition of embryos within egg masses under the epidermis of sorghum leaves makes the embryos accessible for in situ microinjections. (**b**) Egg mass at 2 dpi injected with Cas9 and sgRNAw6-1 and sgRNAw6-2 (n = 17). The egg mass is partially obscured by the leaf epidermis. Melanized injection scars and opaque head caps at the anterior pole are evident. (**c**) A wild-type uninjected egg mass 5 d post deposition (n = 23). The red-brown eye color of wild-type embryos are evident. One embryo did not develop as evidenced by the absence of its headcap. (**d**) Egg mass injected with Cas9 and sgRNAcn4-1 at 5 dpi. The egg mass is partially obscured by the leaf epidermis. Sixteen are *cn* mutants (orange-red), 11 are wild-type (red-brown) and 3 could not be phenotyped but were developing. The powdery white material on injected and non-injected egg masses are brochosomes that are deposited over the egg mass by female GWSS. Embryonic eye colors are reflective of the phenotype of emerging nymphs. (**e**) Egg mass (5 dpi) injected with Cas9 and sgRNAw6-1Syn and sgRNAw6-2Syn (n = 18). Some embryos were not injected to allow easy comparison of the *w* phenotype vs wild-type eye color. Of the ten embryos injected with sgRNAs and Cas9 (#1, 4, 6, 7, 9, 11, 13, 15, 17, and 19 from left to right), 100% were *w* mutants. One embryo did not develop.

We developed a platform for easy and efficient microinjection of GWSS embryos in situ. GWSS females oviposit their eggs side-by-side under the abaxial epidermis of leaves forming an egg mass^24^. Within an egg mass, the anterior to posterior orientation of each egg is identical^25^. In our system, females lay up to 30 eggs/mass (Fig. 1a–e), which is significantly higher than reported values of eight and twelve eggs/mass^24^. Based on the timing of cellular blastoderm in *A. pisum*, *N. lugens* and *O. fasciatus* and absence of any detectable differentiation in GWSS embryos before 90 h post oviposition, we injected GWSS embryos one to two hours after egg deposition^18–20,25^.

To date, the CRISPR/Cas9 machinery has been introduced into Hemiptera by microinjection of embryos removed from the leaf or by injection of the abdomen of gravid females. Our methods are distinct as we inject GWSS embryos in situ. Residing below the plant epidermis, embryo microinjections were simple to perform, as a mass with 20 eggs can be injected within ten minutes by a novice operator. To assess frequency and timing of egg hatch following in situ microinjection, 112 embryos were injected with water. One–two days post microinjection (dpi), a melanized scar developed at the injection site and served as a reliable indicator of embryo viability (Fig. 1b); four-five dpi, embryonic eye spots were clearly visible (Fig. 1c). There was near synchrony in egg hatch with 90.3% of the nymphs emerging at 7 dpi with 64.3% egg to nymph survival (Supplementary Table S1). These data are consistent with earlier reports of GWSS embryo development and nymph emergence on intact plants^25^. In situ injection of embryos on leaves provides an efficient and simple platform for genome modification of GWSS using CRISPR/Cas9 technology.

### High frequency mutagenesis at the *cinnabar* locus

**Figure 2:**
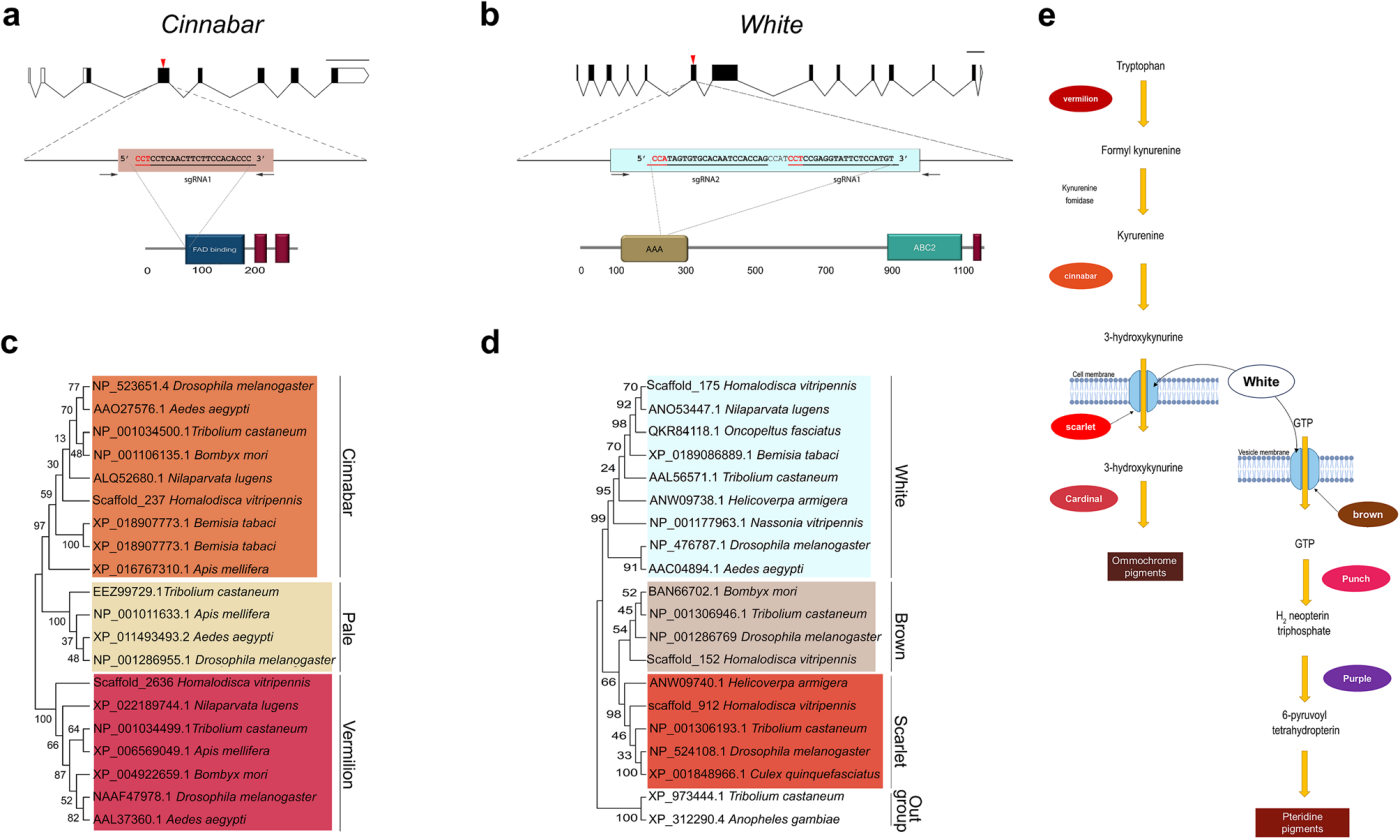
The *cinnabar* and *white* genes of GWSS. (**a**,**b**) The structure of the GWSS *cn* and *w* genes. Exons containing untranslated regions (white bars) and coding regions (black bars), introns (lines), PCR primers (black arrows), and sequence of sgRNA regions (underlined), and PAM sites (red) are shown. The target site of sgRNAs in indicated with a red arrow. Conserved protein domains identified by SMART tool included cinnabar’s FAD-binding (navy) and two transmembrane domains (maroon) and white’s conserved AAA motif (brown), ABC2 (teal), and transmembrane domain (maroon). Scale bars = 1000 bp. (**c**) Phylogenetic tree of cinnabar, pale and vermillion proteins that encode enzymes of the ommochrome pathway (kynurenine 3-monooxygenase, tyrosine 3-monooxygenase, and tryptophan 2,3-dioxygenase, respectively). (**d**) Phylogenetic tree of white, brown and scarlet ABC transporters. (**e**) The ommochrome and pteridine pathways of *Drosophila melanogaster*.

The large red-brown eyes observed in early embryonic development (Fig. 1c) suggested that ommochrome and pteridine biosynthesis pathways control GWSS eye color; these pathways determine eye color in many insects including *Drosophila melanogaster* and the Hemiptera^22,26–28^. Using the new assembly and annotation of the GWSS genome^22^, we identified GWSS orthologs for nine eye-color genes as potential targets. The *cinnabar* (*cn)* gene encodes the enzyme kynurenine 3-monoxygenase, which converts kynurenine to 3-hydroxykynurenin in the ommochrome biosynthesis pathway^29^. The GWSS cn protein was most closely related to cn orthologs from three other hemipteran species and was chosen as a target for CRISPR/Cas9-mediated mutagenesis (Fig. 2a,c,e). sgRNAcn4-1 targets the conserved FAD-binding domain region of *cn* and was used in embryo microinjections with two concentrations of Cas9 (0 and 300 ng/µl) (Table 1). The presence of Cas9 in the injection mix caused a decrease in embryo survival by 31%.

**Table 1:** Injections of Cas9 protein and *cn* or *w* sgRNAs into GWSS embryos.

Gene	sgRNA (300 ng/µl)	Cas9 (ng/µl)	No. expts	No. embryos injected	No. hatched	% hatch	No. with mosaic eye color	% mosaic (to embryos injected)	% mosaic (to hatched embryos)
*cn*	sgRNAcn4-1	0	5	109	87	79.8	0	0	0
		300	7	103	56	54.4	33	32.0	58.9
*w*	sgRNAw6-1 sgRNAw6-2	0	7	142	91	64.1	0	0	0
		150	6	165	90	43.7	72	43.6	80.0
		300	11	290	116	40.0	71	24.5	61.2

**Figure 3:**
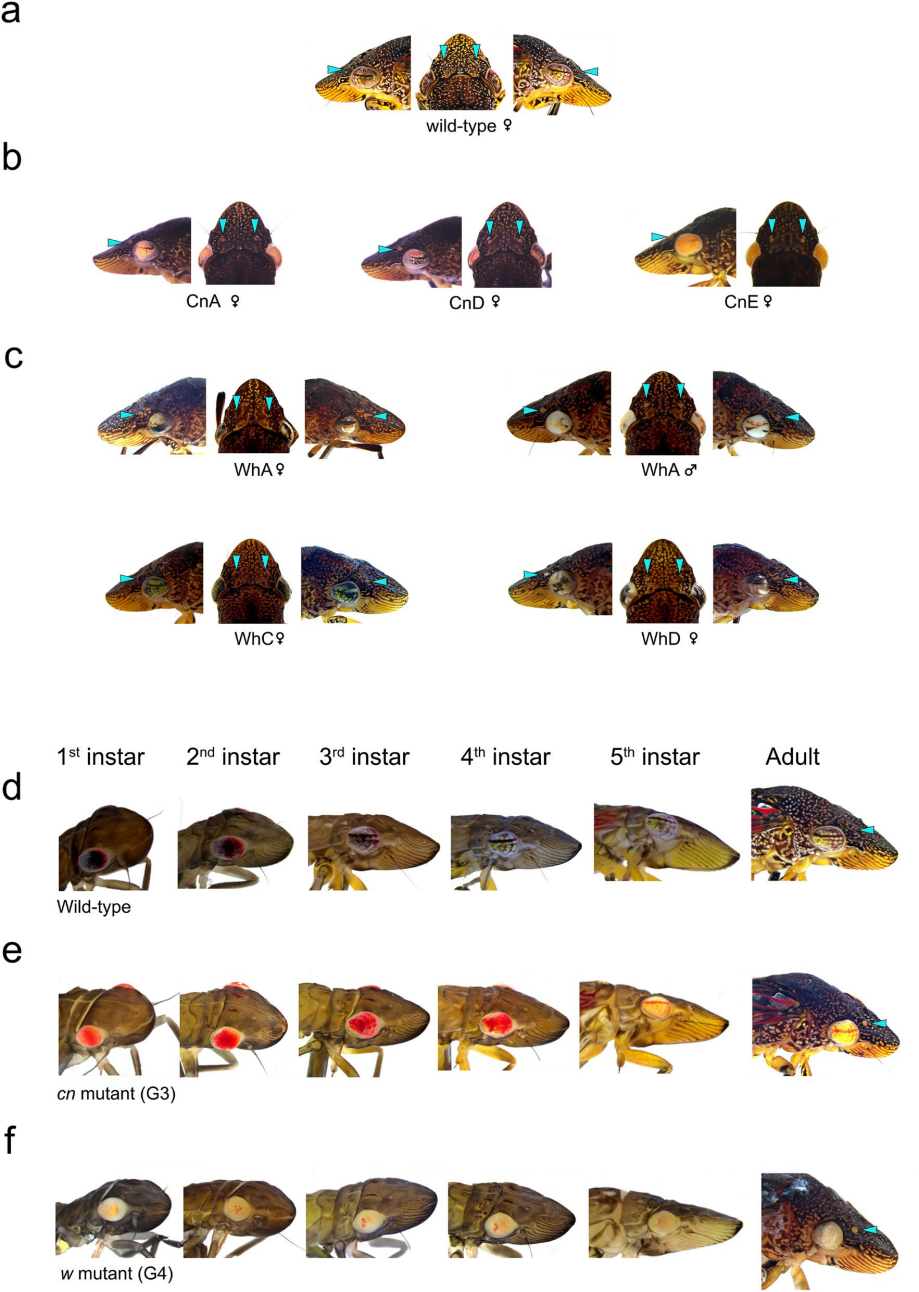
Eye and ocelli of wild-type, *w* and *cn* mutants. (**a**–**c**) Lateral and dorsal views of the adult GWSS eyes and ocelli. (**a**) wild-type female. (**b**) Female *cn* G0 mutants from pooled matings: CnA, CnD, CnE. (**c**) *w* G0 mutants including parents of the WhA line and females of the WhC and WhD lines. (**d**–**f**) Lateral view of eyes and ocelli in 1st to 5th instar nymphs and adults. (**d**) wild-type. (**e**) *cn* G3. (**f**) *w* G4. Ocelli are identified by pale blue arrowheads in the dorsal and lateral views in panels (**a**–**c**).

**Figure 4:**
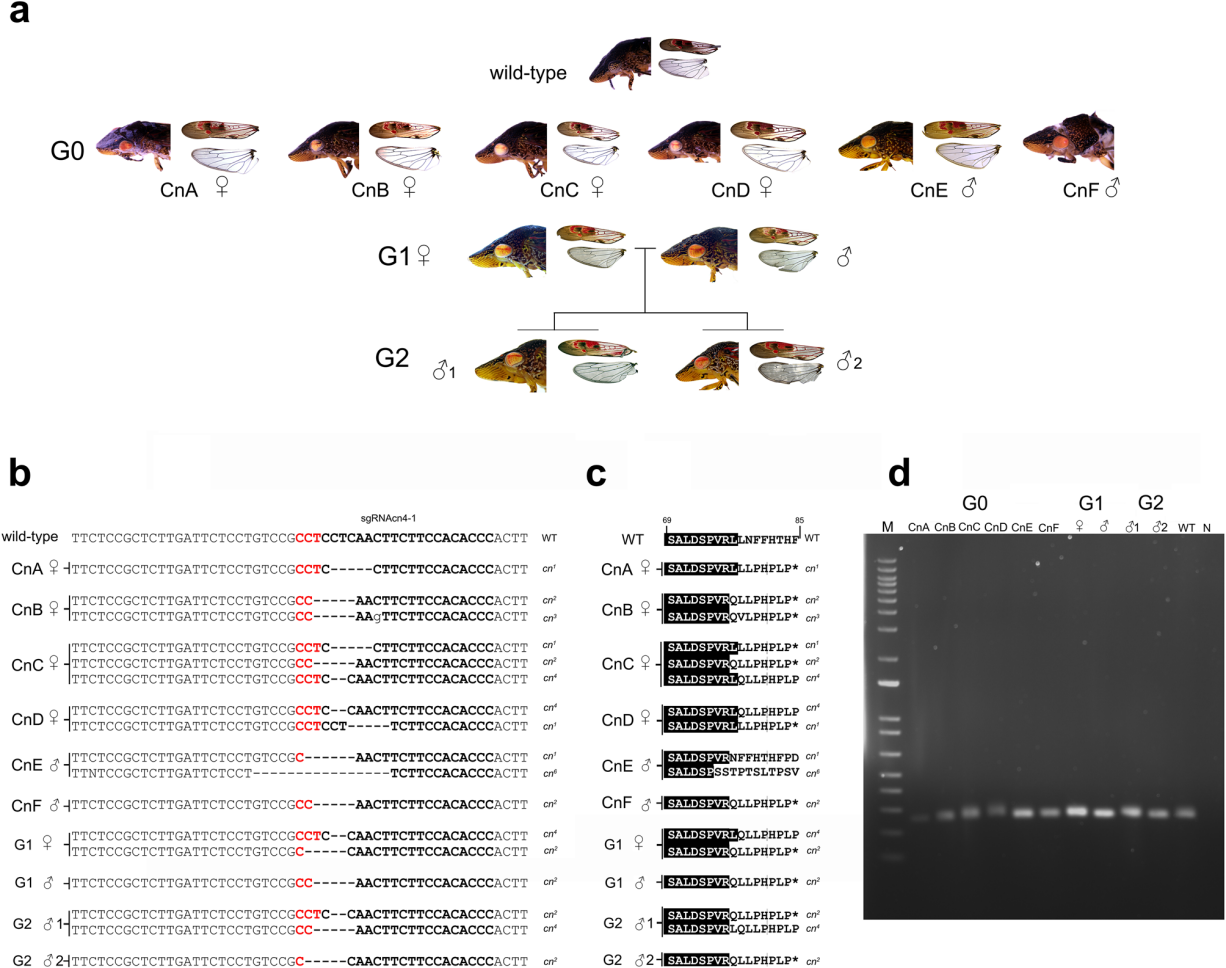
Phenotypes and genotypes of *cn* G0-G2 mutants. (**a**) G0 *cn* mutants were pool mated. One wild-type and six representative G0 *cn* progeny (CnA-F) are shown. The *cn* parents of the G1 cross and two representative G2 progeny are shown. Lateral view of eyes and ocelli, forewings, hindwings and gender are shown for each individual. (**b**) The *cn* target region, location of sgRNAcn4-1, sequence of wild-type and G0-G2 *cn* mutants, and *cn* allele designations are shown. PAM site (red), sgRNA region (bold), and deletions (dashes). (**c**) Deduced amino acid sequences from the wild-type and *cn* alleles spanning residues 69 to 85 of the deduced GWSS cinnabar protein. The full conceptual translation of WT and mutant sequences predicted to produce truncated proteins can be found in Supplemental Fig. 2. Termination codon (*). (**d**) PCR amplification products from the *cn* target region of G0, G1 and G2 insects. Lane N is no DNA template control.

When Cas9 and sgRNAcn4-1 were microinjected into GWSS embryos, we detected G0 late embryos, nymphs and adults with a spectrum of eye colors relative to wild-type GWSS (Figs. 1c,d, 3a,b, 4a). Six representative G0 adults illustrate that the eye-color mosaicism ranged from dark to bright orange with, in some cases, patches of colorless ommatidia (Fig. 4a). Based on phenotypes of G0 adults, the editing frequency was 58.9% (Table 1). In most *cn* G0 mutants, lines of cells with red–orange pigments were organized as arcs across the eye and red–orange ocelli were detected.

Initially, it was not clear if GWSS pair matings would be successful under our rearing conditions. Therefore, to establish *cn* lines, four newly emerged male and eight female G0 *cn* adults were pool mated. Genotypes of six representative G0 individuals (CnA-F) with *cn* mosaic eyes were determined (Fig. 4a–d, Supplementary Fig. S1). PCR amplification of the *cn* target region revealed a single PCR fragment indicating that the editing events did not cause large deletions or insertions (Fig. 4d; Supplementary Table S2). All six *cn* alleles contained small deletions of 5 bp (*cn*^*1*^*, cn*^*2*^*, cn*^*3*^), 2 bp (*cn*^*4*^), 6 bp (*cn*^*5*^) and 16 bp (*cn*^*6*^) at the sgRNAcn4-1 target site consistent with CRISPR/Cas9 mutagenesis (Fig. 4c). All alleles but *cn*^*5*^ resulted in frameshift mutations that are predicted to generate truncated proteins (Fig. 4c, Supplementary Fig. S1). We recovered more than 100 G1 nymphs and identified at least 54 with *cn* eye color. A mutant G1 male and mutant G1 female were selected to demonstrate transmission of *cn* alleles to the G2 generation. Sequencing indicated that the G1 female parent was transheterozygous (*cn*^*2*^*cn*^*4*^*)* and only the *cn*^*2*^ allele was detected in the G1 male (Fig. 4b,c)*.* All G2 progeny had *cn* mutant eyes and sequencing of the *cn* target region indicated that one G2 male was transheterozygous (*cn*^*2*^*cn*^*4*^) and the other was likely homozygous as only the *cn*^*2*^ allele was detected (Fig. 4b,c).

The phenotypes of nymphs and adults in the G3 generation demonstrated that eye pigmentation patterns changed during GWSS development (Fig. 3e). In *cn* mutants, in which the brown ommochromes were not synthesized, the cells accumulating red pigments were revealed. In 1st- and 2nd-instar nymphs, the red-brown and red–orange pigmentation in wild-type and *cn* mutants was homogeneous across the eye, respectively. In later instars, patterning of pigments was different between wild-type and *cn* mutants. The lines of cells that form prominent brown horizontal stripes and pigmented arcs across the eyes were detected in the 3rd instar to adult in wild-type GWSS. In contrast, in *cn* mutants the horizontal pigment stripes were detected later and only in eyes of the 5th instar and adults, whereas the pigmented arcs were detected in the 3rd–5th instars and persisted through adulthood (Fig. 3e).

### High frequency mutagenesis at the *white* locus

Using the new assembly and annotation of the GWSS genome^22^, we identified GWSS orthologs for the white, scarlet and brown proteins, which are ABC transporters that import pigment precursors into the developing eye (Fig. 2e). In *D. melanogaster*, the white (w) protein heterodimerizes with either the scarlet or brown proteins to import ommochrome and pteridine precursors, respectively^30,31^. Two sgRNAs within exon 6 of the *w* gene that target the conserved AAA domain were designed^32^ (Fig. 2b). Phylogenetic trees indicated that the GWSS w protein was most closely related to w from three other hemipteran species and more distantly related to the brown and scarlet proteins (Fig. 2d).

To determine optimal conditions for editing the *w* gene, GWSS embryos were injected with two *w* sgRNAs (sgRNAw6-1 and sgRNAw6-2) and different amounts of Cas9 (Table 1). Relative to the zero Cas9 control, Cas9 (150 and 300 ng/µl) decreased embryo survival by 20.4% and 24.1%, respectively (Table 1). A higher frequency of mutagenesis was achieved with the lower Cas9 concentration. At four-five dpi, eyes were evident and developing embryos with wild-type and *w* mutant eye colors were easily discernible by the naked eye (Fig. 1e). These embryonic phenotypes were confirmed in G0 mutant nymphs and adults, where the degree of eye color mosaicism varied between individuals (Figs. 3c,f, 6a). In addition to mosaic eyes, G0 adults with strong *w* phenotypes had white ocelli.

We established four independent crosses (WhA-D) with pools of male and female G0 adults with mutant eye color (Figs. 3c, 6a). While all four crosses produced egg masses, only WhA, WhB and WhD produced nymphs; the WhC eggs did not hatch and were likely unfertilized. In WhA, we observed a mating couple and, following copulation, the male was collected for genetic analysis and the female placed in isolation for the establishment of the WhA line. Given that this line may be the result of a single-pair mating, we focused on the maintenance and genetic analysis of this line through subsequent generations.

The G0 female parent used to establish the WhA line had dark mosaic eyes and its mate had white eyes with a distinctive mosaic pattern and white ocelli (Figs. 3c, 6a). Mutant eye color was observed in G1 embryos at four-five dpi, in all instars, and the G1 through G4 adults (Figs. 3f, 6a). In eyes of some G1 *w* progeny, residual amounts of red pteridines were detected against a primarily white background; the red arcs mimicked the brown striations seen in wild-type eyes (Fig. 6a). The white eyes and ocelli were transmitted for four successive generations (Fig. 3f). In total, 24 mutant and nine wild-type G1 progeny were obtained from the WhA G0 cross.

**Figure 5:**
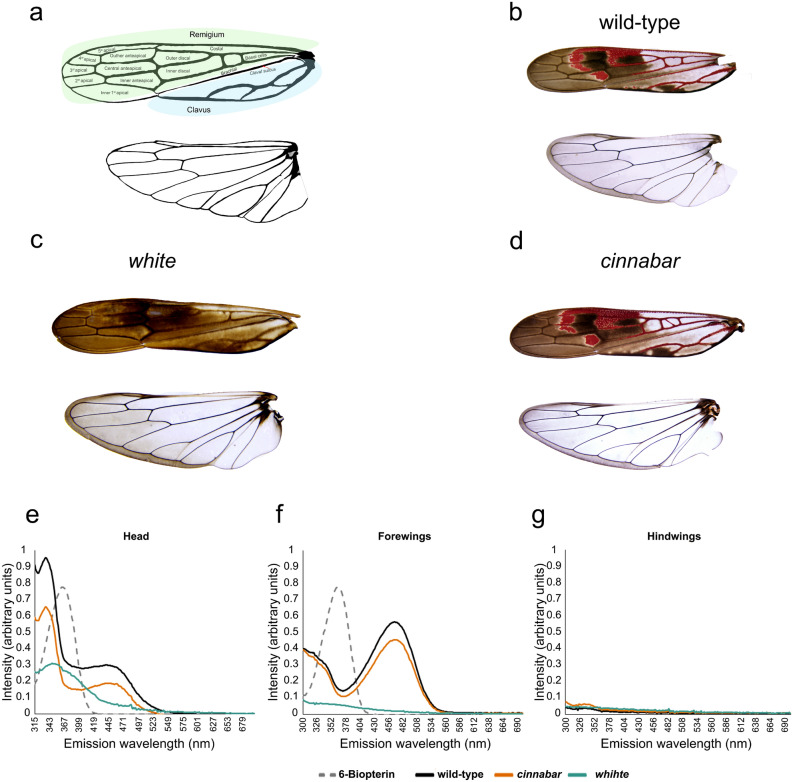
Wings of wild-type, *w* and *cn* mutants. Forewings and hindwings (**a**) Line drawing of the forewing and hindwing of GWSS with interveinal spaces labeled. The remigium is shaded pale green and the clavus pale blue. (**b**) wild-type. (**c**) *w* G4. (**d**) *cn* G3. Forewings (upper) and hindwings (lower) are displayed in each panel. The forewing’s remigium and clavus are separated by the claval suture located immediately below the brachial interveinal space^61^. (**e,f**) Pteridine pigments. Pteridines were extracted from (**e**) heads, (**f**) forewings**,** and (**g**) hindwings of wild-type, *w* and *cn* GWSS. Tissue extracts were spectrophotometrically assessed for pteridines by scanning from 300  to 690 nm. 6-biopetrin was used as a standard.

**Figure 6:**
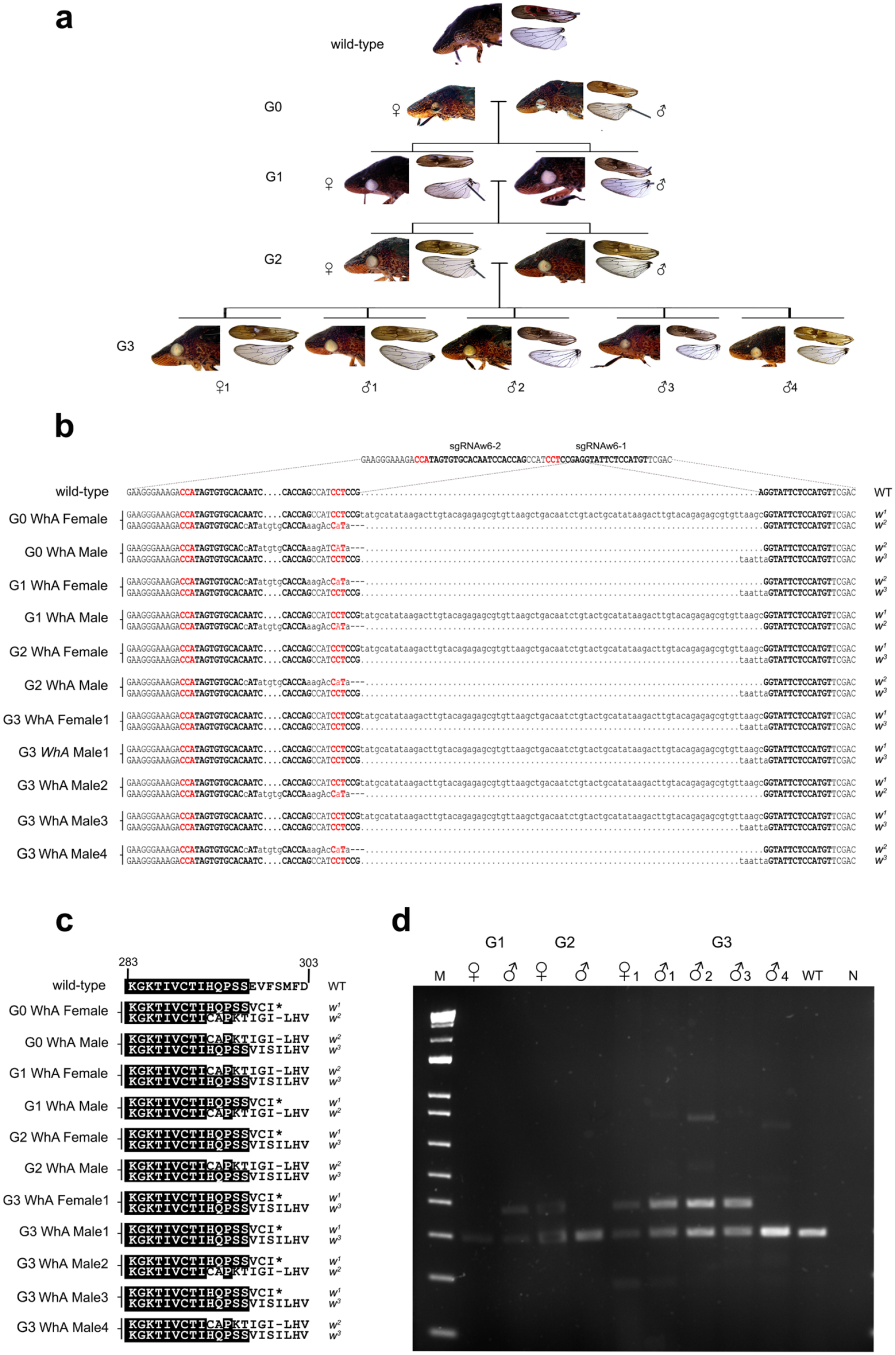
Phenotypes and genotypes of *w* G0-G3 mutants. (**a**) Parents of the WhA line, parents of the WhA G1 and G2 crosses, and five representative WhA G3 progeny. Lateral view of eyes and ocelli, as well as forewings and hindwings, are shown for each individual. (**b**) The *w* target region, location of sgRNAw6-1 and sgRNAw6-2, sequence of wild-type and G0-G3 *w* mutants, and *w* alleles are shown. PAM site (red), sgRNAs (bold), insertions and substitutions (lower case), deletions (dashes). (**c**) Deduced amino acid sequences from the wild-type and *w* alleles spanning residues 283 to 303 of the GWSS white protein. Residues identical to the wild-type protein are highlighted in black. Termination codon (*). (**d**) PCR amplification products from the *w* target region. The 83-bp insertion of the *w*^*2*^ allele is easily resolved. Lane N is no DNA template control.

In all generations (G0-G3), *w* mutant adults displayed notable differences in the color of their forewings relative to wild-type insects (Figs. 5a–c, 6a). The hindwing of GWSS was unpigmented with the exception of brown pigments (likely melanins) in the marginal regions. In contrast, as previously noted^33^, the forewing possesses distinctive red pigmentation of the veins and interveinal spaces superimposed on the brown pigmentation of the forewing*.* Red pigments were detected in the partially confluent veins of the clavus (Fig. 5b). In addition, red veins flanked the interveinal spaces in the basal portion of the remigium but not in the veins surrounding the anteapical and apical regions of the forewing. Red pigments were also detected in the interveinal spaces including the basal portion of the inner anteapical and 5th apical spaces, the central portion of the central and outer anteapical spaces, and the apical portion of the outer discal and costal spaces; all of these red-pigmented regions were surrounded by a white margin (Fig. 5b). In *w* mutants, the red pigments of veins and the intervening spaces were absent and white unpigmented regions replaced the red domains (Fig. 5c).

To determine if the GWSS red pigments were pteridines, pteridines were extracted from forewings, hindwings and heads of wild-type, *w* and *cn* mutants (Fig. 5e–g). Pteridines were at very low to undetectable levels in wild-type, *w* and *cn* hindwings. In contrast, two classes of pteridines (with peak absorbances at 334 nm and 467 nm) were detected in heads and forewings. In both wild-type and *cn* GWSSs, the 334-nm pteridines had an absorbance close to 6-biopterin (standard), one of the most common pteridines in insects^34–36^, and were more abundant in heads than forewings. In contrast, the 467-nm pteridines were more abundant in GWSS forewings (Fig. 5f). In *w* mutants, the 334-nm and 467-nm pteridines were reduced in the head. Even more striking was the minute quantities of both pteridine classes in forewings of the *w* mutant (Fig. 5f). These data suggest that the red pigments of the GWSS wings were pteridines.

To evaluate the number of mutations (inherited and not inherited) in G0 mosaic insects, we constructed *w* target-site amplicon libraries from the female and male parents of the WhA line, single G0 females from the G0 crosses WhB, WhC and WhD, and a non-injected, WT female. High rates of mutagenesis were detected in the G0 insects ranging from 11% (WhC female) to 99.94% (WhD female) (Supplementary Table S3). Mutations detected were consistent with Cas9 cleavage 3 bp upstream to the PAM sites and repair of the dsDNA breaks by non-homologous end joining (NHEJ) (Fig. 3, Supplementary Fig. S2a–f, Supplementary Table S3). In all five G0 GWSS adults sequenced, *w* sgRNAw6-1 was more efficient than sgRNAw6-2 in generating mutations. The number of unique *w* alleles detected in the five G0 mutant adults ranged from 227 to 1113 (Supplementary Table S3c). Deletions predominated being twice as frequent as insertions and > tenfold more frequent than substitutions (Supplementary Table S3). In contrast, the WhC female had low mutagenesis rates and substitutions predominated (Supplementary Fig. S2e).

To assess the parental origin and inheritance of the *w* alleles in insects derived from line WhA, we determined the sequence of the *w* target region in selected G1, G2 and G3 individuals, which were generated by single-pair matings (Fig. 6a). Three alleles (*w*^*1–3*^*)* were detected in the WhA lineage (Fig. 6b,c). The G0 female parent carried the *w*^*1*^ allele, which was an 83-bp insertion that began 3 bp upstream to the PAM site adjacent to the sgRNAw6-1 target; this generated a stop codon at residue 300, which would be predicted to produce a truncated and likely nonfunctional w protein (Fig. 6c, Supplementary Fig. S1). The second allele (*w*^*2*^) in the G0 female parent and G0 male parent had a 4-bp insertion 14 bp upstream to the PAM site adjacent to the sgRNAw6-2 target and a 3-bp deletion adjacent to the sgRNAw6-1 PAM site, as well as seven substitutions. The net outcome was a frameshift likely to result in a truncated peptide 323 amino acids (aa) in length (Fig. 6c, Supplementary Fig. S1). The *w*^*3*^ allele from the G0 male parent contained a frameshift due to a 4-bp insertion and 2 substitutions located 3 bp from the sgRNAw6-1 PAM site; this frameshift at aa 297 would be predicted to produce a protein prematurely terminated at residue 324 (Fig. 6c, Supplementary Fig. S1). Based on *w* target region sequence analysis and PCR products, all the G1, G2 and G3 *w* individuals carried two *w* mutant alleles, which was consistent with Mendelian inheritance of these three alleles (Fig. 6b–d, Supplementary Fig. 4). This is also consistent with the white eye color and the absence of red pigment in the wings, suggesting dysfunction of the proteins from the *w*^*1*^*, w*^*2*^*,* and *w*^*3*^ alleles (Figs. 5, 6a). The residual red-pigmented spots that were seen within eyes of some *w* mutants suggested that one or more of these proteins may be partially functional in selected cells within the GWSS eye (Figs. 3c,f, 6a).

### The *w* and *cn* genes are located on autosomes

**Figure 7:**
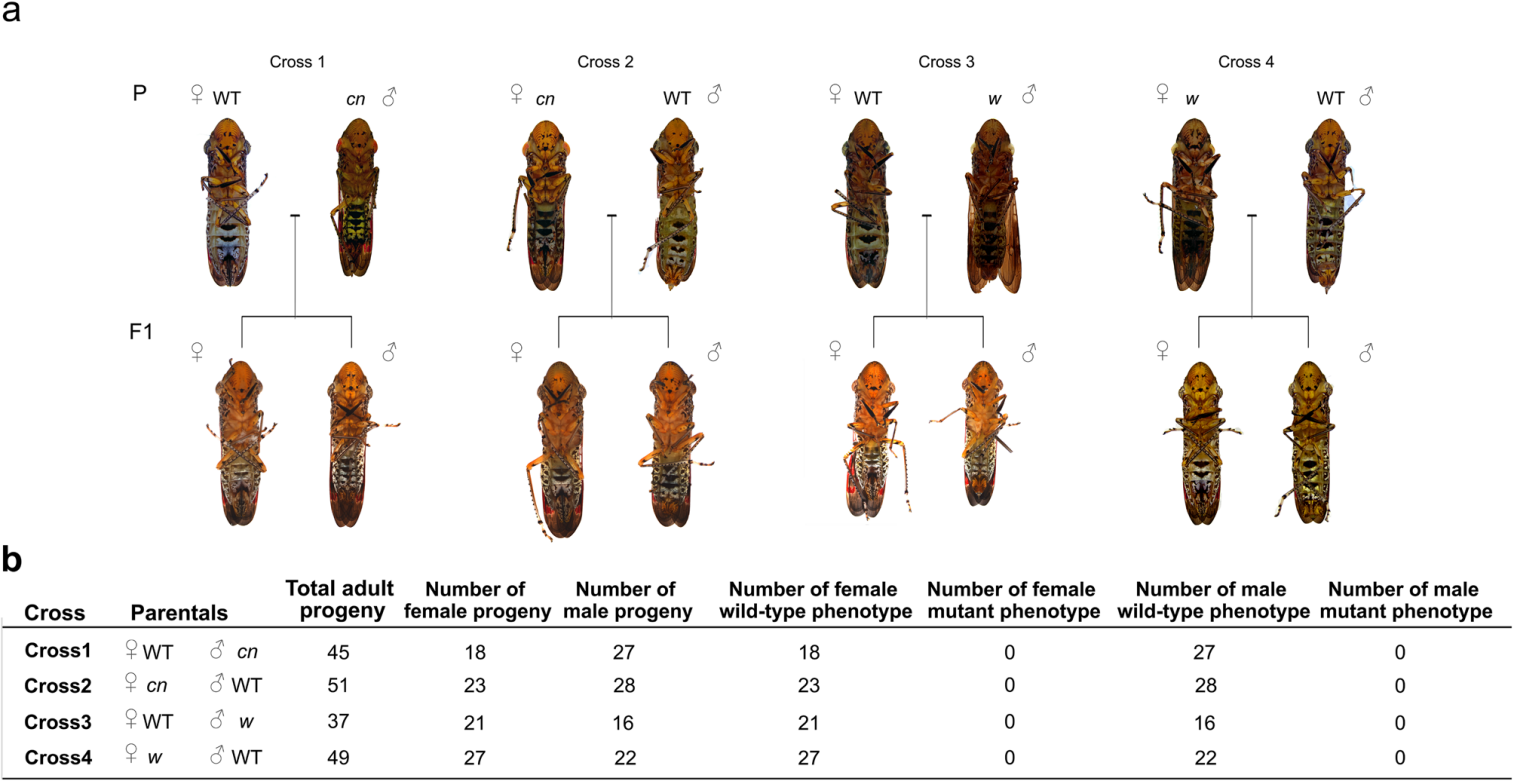
*w* and *cn* are autosomal. Reciprocal crosses of wild-type (WT) with a *w* mutant and WT with a *cn* mutant. (**a**) Ventral view of GWSS adults and two representative progeny. The parents and representative F1 progeny from crosses 1, 2, 3, and 4 are shown from left to right. (**b**) Number of adult F1 progeny, their gender and eye-phenotypes from crosses 1, 2, 3, and 4.

Our ability to establish *w* and *cn* lines and to perform pair matings with GWSS allowed us to perform reciprocal matings between mutant and wild-type adults to directly determine if *w* and *cn* were sex-chromosome linked or reside on autosomes. Four crosses were performed using *w* or *cn* mutants as male or female parents in crosses with wild-type insects (Fig. 7a). F1 progeny were phenotyped for eye color and sexed. All F1 progeny were wild-type indicating the recessive nature of the *w* and *cn* alleles and consistent with the location of both *w* and *cn* on autosomes (Fig. 7b). If either *cn* or *w* were X-chromosome linked, males would have inherited the mutant allele from their mothers in crosses 2 and 4 and displayed a mutant eye-color phenotype. These data were further supported by our genotypic data, acquired in parallel, that demonstrated two *cn* alleles and two *w* alleles were present in both sexes of G1, G2 and G3 adults (Figs. 4, 6).

### Off-target analysis using the *w* and *cn* gRNAs

**Figure 8:**
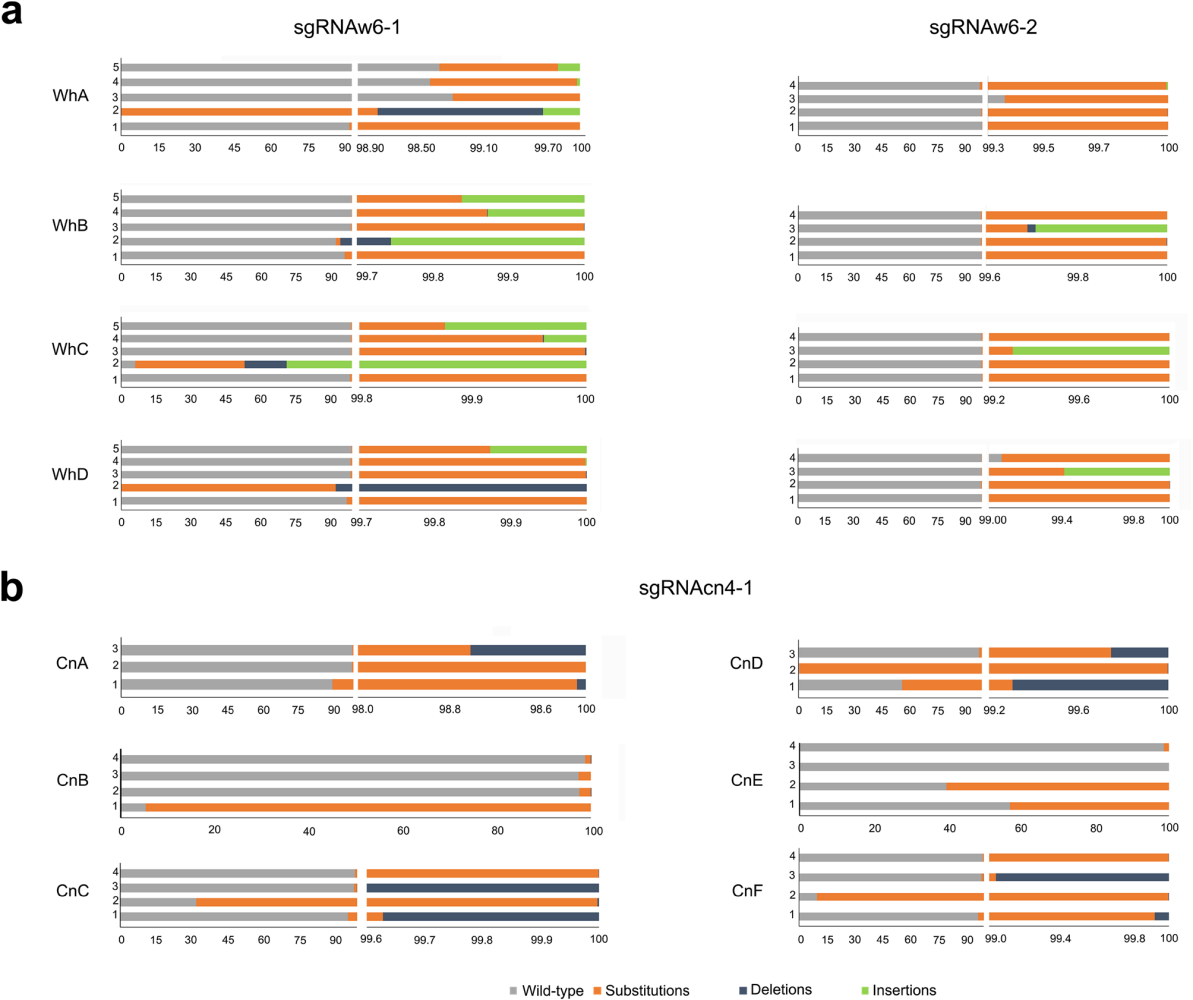
Off-target analysis of the *w* and *cn* sgRNAs. Putative off-target sites for the (**a**) sgRNAw6-1 and sgRNAw6-2**,** and (**b**) sgRNAcn4-1 were identified (Supplementary Table 4). Mutations at these sites were assessed by amplicon sequencing in G0 GWSS adults. The percent of wild-type sequences (grey), substitutions (orange), deletions (blue), and insertions (green) were determined. The inheritance of off-site mutations was not assessed. Note the breaks in the x-axes when mutations were detected in less than 1% of reads.

Genetic analysis and genetic control strategies require that mutations generated by CRISPR/Cas9 gene-editing strategies be target-site specific. Given the exceptionally high rates of CRISPR/Cas9-editing in GWSS, it was critical to assess sgRNA specificity in vivo. Cas-OFFinder was used to identify potential off-target sites^37^. Four to five off-target sites for *w* and *cn* were chosen for analysis. Amplicon libraries for each off-target were prepared from genomic DNA from WhA-D or CnA-F G0 females. Of the 11 libraries analyzed, mean % reads mapped to the off-target ranged from 0 to 0.95%; however, sgRNAw6-1’s off-target 4 had a larger % reads (5.04%) (Fig. 8, Supplementary Table S4). These data indicated that off-target editing did not occur or occurred infrequently. Our data are comparable to the negligible off-target frequencies from *Anopheles gambiae* in which the impact of off-target effects on a gene drive strategy was determined^38^.

## Discussion

We report the establishment of genetic analysis in GWSS, a significant hemipteran pest of agricultural crops in North and South America. Several factors enabled us to establish this genetic platform. First, was access to the newly assembled and annotated GWSS genome^22^, which identified target eye-color genes and off-target genes to assess specificity of mutagenesis. Second, was use of leaf segments as a physical platform for in situ microinjection of GWSS embryos through the leaf epidermis and their development to hatching. Third, was the use of beveled quartz needles that cleanly pierced the leaf epidermis and embryo inflicting little damage to either organism. Fourth, was the ability to perform pair matings of GWSS. Our ability to break the winter diapause now allows for year-round genetic manipulation and analysis of this pest insect. Finally, our system provided an early read-out of editing frequencies. At five dpi, the embryonic eyes were visible, allowing easy discrimination of mosaic-eyed G0 mutants from wild-type embryos before egg hatch.

We were able to routinely generate high frequency mutations at the *w* and *cn* loci. For example, G0 *w* mutants were generated at frequencies between 61.2 and 80.0% with low rates of off-target mutations. Pair matings allowed us to establish a *w* line and follow the inheritance of three *w* alleles to the G3 generation. There was 100% transmission of the *w* alleles and G1, G2 and G3 individuals transheterozygous for two mutant *w* alleles were recovered. The *w* gene has been previously used in proof-of-concept experiments in five other hemipteran species: corn planthopper (CPH), neotropical stink bug, brown planthopper (BPH), milkweed bug, and whitefly^12,14,16,19,20^. The rates of CRISPR/Cas9 mutagenesis based on eye phenotypes of G0 insects varied significantly from 0.2–2.5% (whitefly), 0–27.3% (BPH), 32.4% (CPH), 33% (stinkbug) and 14.0–92.5% (milkweed bug). Significantly, GWSS mutation rates were at the high end of this spectrum of mutation efficiencies. These differences could be due to differences in locations and numbers of sgRNAs used and also in the methods used for macromolecule delivery.

Significant fitness costs of *w* mutations have been reported in other Hemiptera. For example, G1 insects with *w* mutant alleles could not be generated in CPH and stinkbug^12,16^. Similarly, transmission of *w* alleles to the G1 generation in BPH and whitefly was low (3% and 1%, respectively)^14,20^. Finally, while 65% transmission of *w* alleles to the G1 was reported in milkweed bug, lines with two* w* mutant alleles could not be established*.* Collectively, these data suggest that a functional w protein is required for the viability of these hemipteran species^19^; alternatively, few *w* alleles were in the germline cell lineage of these G0 insects. To date, *cn* mutations have only been reported in *N. lugens*^20^. While none of the G0 *N. lugens* adults displayed a *cn* phenotype, 49% of G1 insects from pair matings had the red eyes indicative of *cn* mutant alleles^20^. In contrast, in GWSS, *cn* mutagenesis was 58.9%, there was 100% transmission of *cn* alleles to the G1 and G2 generations and insects carrying two mutant *cn* alleles and possible homozygous *cn* mutants were identified.

The analysis of the GWSS *w* and *cn* mutants provided significant insights into the pigmentation of Cicadellidae eyes, ocelli and wings. The w protein transports precursors of the ommochrome and pteridine pathway; therefore, mutations in *w* often result in the total absence of eye color^26^. In contrast, since *cn* only impacts the ommochrome pathway, the tissues with pteridines will be revealed. The location of these pigments can differ between insect orders^26,28,36,39^. Within the Hemiptera, ommochromes and pteridines are confined to the adult compound eye and epidermis, respectively^26^. More recently, pteridines were detected in water strider embryonic eyes and epidermis^28^. Our results with GWSS concur with these findings, as pteridines were detected in GWSS eyes, ocelli and forewings (epidermis) of adults and eyes of embryos and nymphs.

Timmons et al.showed that the red pigments of the GWSS forewing fade during adulthood and they speculated that pheomelanin was the red pigment^33^. However, our discovery that red pigments were absent in the veins and interveinal spaces of the forewings of *w* adults indicated that pteridines are the red pigments in GWSS wings. The spectral analysis of pteridines in the heads and wings of GWSS revealed a surprising complexity with two pteridine forms present in these organs. The 334-nm and 467-nm pteridine forms were most abundant in the GWSS heads and forewings, respectively, of wild-type and *cn* mutants. While the chemical identity of the pteridines within GWSS eyes and wings are currently unknown, the red-pigmented erythropterins occur in the cuticles of several hemipteran species^40,41^ and this pteridine has a spectrum similar to the 467-nm pteridine in pierid butterfly wings^42^. The 334-nm pteridines may be more related to colorless 6-biopterin^36^. Whether the wing pteridines have a role as a warning signal for GWSS to avian predators, as occurs for some heteroptera, is currently unknown^43^. Furthermore, the retention of the brown pigments in the GWSS forewings and hindwings in both *w* and *cn* mutants suggest that ommochromes are not the brown pigments in GWSS wings. These data implicate melanins as the brown pigments in GWSS wings, as previously suggested^26,33^. Finally, the white halo that surrounds the red zones of WT forewings and the white zones in *w* forewings, indicates that cells in these “halo” regions follow a different developmental program than the surrounding cells that express eumelanin.

The absence of eye color in the G1, G2 and G3 individuals of the *w* line of GWSS is consistent with previous work documenting ommochromes in hemipteran eyes^12,16,19,20,26^. Furthermore, the red–orange eyes and ocelli of GWSS *cn* mutants are consistent with the discovery of pteridines in water strider and BPH eyes^20,28^ and also indicate that the yellow and brown pigments seen in the wild-type eyes and ocelli do not result from pteridines but are most likely due to ommochrome pigments. It is also noteworthy that the mosaicism of GWSS *cn* eyes was also seen in the mosquito *Aedes aegypti*, where *cn* is cell autonomous; however, mosaics were not detected in *Drosophila,* where *cn* is not cell autonomous, nor was it recorded in BPH^20,44^.

Unlike other species studied to date, eye color patterning in GWSS eyes is complex. The eyes of GWSS 3rd–5th instar nymphs and adults have strong horizontal stripes and brown pigmented arcs. In the *cn* mutants, these patterns were recapitulated by cells expressing pteridine pigments; but, the appearance of the horizontal stripes was delayed in the *cn* nymphs. In the *w* mutants, the ommochrome and pteridine patterning is largely abolished; although, residual amounts of red pigment can be detected as punctate spots and occasionally as discontinuous arcs in the eyes of *w* nymphs and adults. These data suggested that the *w* mutant proteins were partially functional and pteridines accumulated to sufficient levels in a small subset of cells in the GWSS eye. Alternatively, GWSS eyes expressed an unknown red pigment in a subset of eye cells. The importance of these pigmentation patterns in GWSS biology and eye function are not understood. It is well established that both ommochromes and pteridines are secondary eye pigments that help capture light that is not directly absorbed by the ommatidia^45^. However, the importance of eye color patterns in visual acuity, behaviors, or as camouflage have yet to be resolved^43^. Our stable mutant lines will allow us to approach these fundamental questions in the Cicadellidae.

Within the Hemiptera, three types of chromosomal sex determination systems have been documented: XX/XY, XX/XO, and haplodiplody^46^, While the chromosomes of GWSS have not been directly observed, the cytogenetic analysis of 25 species from the Cicadellidae and genomic and transcriptomic analyses suggest that the Cicadellidae have an XX/XO system of chromosomal sex determination^47,48^. We were able to unequivocally demonstrate that *w* and *cn* are located on autosomes; furthermore, genotyping of both *w* and *cn* mutant individuals from G1, G2 and G3 indicated that both males and females carried two alleles for each of these genes. This contrasts to the X-linked inheritance of *w* in *D. melanogaster*^49^, but is consistent with the autosomal location of *w* in the hemimetabolous house cricket, *Acheta domesticus*^50^. Given the conservation of X-linked genes in three hemipteran insects (*O. fasciatus*, the brown marmorated stink bug (*Halyomorpha halys)* and GWSS) with different sex determination systems (XX/XY, likely male heterogametic*,* and XX/XO, respectively*),* it will be interesting to determine if the autosomal location of *w* will be maintained across the Hemiptera^48^.

Our data illustrate that GWSS provides a robust platform for genetic manipulation in Hemiptera and, given our high frequencies of knock-out mutagenesis, GWSS holds promise to emerge as a model genetic system of the Hemiptera^23^. The development of CRISPR-based methods for gene insertion (knock-in mutagenesis) in this pest is feasible, even if a large decrease in the efficiency of gene insertion occurs in GWSS; our preliminary data suggest that knock-in mutagenesis can be achieved with a relatively small number of embryo injections. With the tantalizing possibility of gain-of-function mutagenesis in the near future, our challenge is to generate cogent genetic control strategies that will prevent the economic damage caused by GWSS’ voracious feeding and transmission of *X. fastidiosa* to uninfected plants.

## Methods

### Biological materials

All local, national and international guidelines were adhered to in this research.

#### Host plants

All plant species used were commercially available: basil (Ocimum basilicum, Italian large leaf seeds, Lot# N6159, http://www.EdenBrothers.com), okra (Abelmoschus esculentus, cow horn seeds, Lot#27435, http://www.EdenBrothers.com), sorghum (Sorghum bicolor, grain red milo seeds, Lot# 200427022009, http://www.twowilliesnursery.com), and sunflower plants (Helianthus annuus)\,peredovik sunflower seeds, Lot# SUNN01N AMS #3427, http://deercreekseed.com) were used for colony and insect line maintenance, microinjection experiments and insect crosses. All plants were grown in greenhouses under a 14:10 h light:dark cycle using supplemental high-pressure sodium bulbs and temperatures ranging from 24 to 30 °C (+/– 5 °C). The plants were grown in 2-h steam-treated UC Riverside soil mix 3 and fertilized weekly with Miracle-Gro All Purpose Plant Food fertilizer. Three- to-five-week-old plants were used for wild-type and mutant GWSS colony maintenance. For collection of egg masses for microinjections, 2-to-8-week-old sorghum was used.

#### *Homalodisca vitripennis* colony

Wild *H. vitripennis* Germar populations were collected as adults in Kern County, California, in August 2019. Seventy-five to one hundred-thirty insects were placed in BugDorm cages (60 × 60 × 60 cm; Bioquip, California, USA) and kept in a greenhouse with a 14:10-h light:dark cycle with temperatures ranging from 24 to 30 °C (+ /− 5 °C). Wild-type and mutant lines of GWSS were provided a diverse host plant diet, which included basil, okra, sorghum, and sunflower plants in a 1:2:1:2 ratio. Plants in colonies were replaced with fresh plants every one to 2 months depending on cage population density. Insect populations were maintained with 75 to 130 insects per cage. Mutant colonies and lines were maintained under ACL2 insectary containment. The colonies were maintained at 14:10-h light:dark cycle, 24–28 °C, and relative humidity of 35% to 50%. Under these growth conditions, the wild-type and mutant colonies produced gravid females continuously throughout the year.

### Identification of *cinnabar* and *white* target genes and design of sgRNAs

Drosophila and *Bemisia tabaci* w and cn proteins and BlastP were used to identify the orthologs in the GWSS genome version 1.0 (https://i5k.nal.usda.gov/webapp/blast)^51^ and a 2021 GWSS genome assembly^22^, respectively. For the phylogenetic analysis, w and cn protein structures were predicted using SMART tool. Phylogenetic trees were constructed based on the deduced amino acid of each gene by the maximum likelihood method combined with poison model for amino acid substitution using MEGA X tool^52^. Numbers in the tree indicate bootstrap values (1000 replicates). Sequences were retrieved from the GenBank database. Based on Drosophila w protein structure, *w* guides were designed in the region of the ABC-transporter-like domain. The sgRNAs were designed using the CHOPCHOP webtool^53^. Potential nonspecific targets were identified by alignments (BlastN) between candidate sgRNA sequences and i5K genome^22,54^; sgRNAs with nonspecific targets were eliminated from consideration. sgRNA sequences are found in Supplemental Table S5.

### Embryo microinjections

Gravid females in wild-type GWSS colonies were identified by white brochosome deposits on their forewings and were transferred to a sunflower plant and confined using bread-bag-cages (9″ × 4″ × 13″ polypropylene bread bag, PJP Marketplace, Philadelphia, USA). The infested plants were transferred to a growth chamber for 24 h. One or two gravid females were subsequently transferred to a single bread-bag-enclosed sorghum for oviposition. The plant was checked every hour for egg masses. Sorghum leaves with egg masses were excised from the plant and each leaf segment placed on 6-cm petri plate containing 1% Gamborg’s B-5 phytoagar with vitamins (Sigma). Embryo microinjections occurred 1–2 h after oviposition. The angle of attack was between 35° and 50° and the D-axis of the micromanipulator control used for penetrating and withdrawing the needle though the leaf epidermis and the embryonic chorion and internal membrane. Microinjection was anywhere from the middle to the posterior of the embryo, with the posterior end being at the opposite end to the oviposition scar. Injection pressures varied between embryos but were typically in the range of 107–348 HPa (1.61–5.22 psi) with transfer pressures typically in the range of 82–210 HPa (1.23–3.15 psi). The Injection mix could be seen entering the embryos.

The plates containing the microinjected embryos were covered with mesh lids, sealed with parafilm and incubated (28 °C, 14:10 light:dark cycle, 60% humidity). The leaf segments with eggs and agar were curated daily using a cotton swab and scalpel to remove any fungus or decaying leaf tissue. The plates were resealed and returned to the incubator. Developing embryos were screened daily for eye-disc color (see below). Nymphs emerged between days 7–10 post injection.

### Establishment and maintenance of GWSS edited lines

To establish edited GWSS lines, 8-day-old adults with *w* or *cn* mutant eyes were sexed based on genitalia features as described by Hummel et al.^55^, and single-pair matings were established. For the *w* line WhA, a single G0 virgin female and G0 male were reared on six 2- to 4-week-old sunflowers^56^. Cages were monitored daily until there was evidence of oviposition (G1 generation). After ~ 100 G1 nymphs had developed into adults, the G0 parents were sacrificed, photographed, and stored at − 80 °C for molecular analysis.

To generate the G1 generation of the *w* line WhA, three pair matings were set up. Both female and male G1 parents had *w* eye-color phenotypes. The G2 and G3 generations of line WhA were established from one of the three G1 pair matings. The G4 generation was established from a G3 pair mating. The remaining G1 insects with mutant eye phenotypes were pooled together (pool mating) and maintained as described above of the wild-type and mutant GWSS colony.

For the *cinnabar* line, 12 G0 individuals were confined to a cage containing a 1:2:1:1 plant ratio and kept until there was evidence of oviposition (G1 generation). After the G1 nymphs emerged, the G0 parents were sacrificed, photographed, and stored at − 80 °C for molecular analysis. When red-eyed *cn* G1 adults emerged, three pair matings were established. One G1 pair was selected to give rise to the G2 generation using the pair-mating protocol. Cn colonies with subsequent generations were maintained with pool matings.

### Inheritance of *white* and *cinnabar*: reciprocal crosses

To determine if *w* and *cn* loci were autosomal or sex-linked, four independent crosses were made: (1) WT female X *cn* male; (2) *cn* female X WT male; (3) WT female x *c*n male; and (4) *w* female X WT male. Crosses were established using G3 *w* parents and G0/G1 *cn* parents. For each cross (pair mating), a virgin female and a male were placed in individual cages for 2–4 weeks; each cross was replicated three times. If a parent died prior to F1 nymph emergence, a new insect was added to the cage. The adult F1 progeny (n > 51) were assessed for eye color, wing pigmentation and gender.

### Off-target identification

Off-target sites were identified by interrogating the 2021 GWSS genome assembly using the Cas-OFFinder tool^22,37^ (Supplemental Table S4). Cas-OFFinder was run with the following parameters: mismatch ≤ 4, DNA bulge ≤ 2, RNA bulge ≤ 1. Off-target candidates were filtered to identify sites within an intron or exon and had an exact match to the PAM site and seven bp of the seed region adjacent to the PAM site. The Cutting Frequency Determination (CFD) score of Doench et al.^57^, which assesses the likelihood of Cas9 cutting at a site, was used to rank the potential off-targets. Finally, we focused on off-targets with nucleotide polymorphisms or bulges furthest away from the seed region^58,59^, as they would be the most likely off-target sites. Four-to-five off-target sites were selected for each sgRNA (sgRNAw6-1, sgRNAw6-2 and sgRNAcn-4) and primers designed (Supplemental Table S4).

### Sequence analysis of *w* and* cn* target loci in G0-G3 insects and off-target loci in G0 insects

G0 insects are genetic mosaics and only a subset of the mutations detected in G0 insects (those in germline cell lineages), will be inherited; however, assessing the genetic diversity in G0 insects provides a rigorous assessment of efficiency of a sgRNA in editing and its specificity (as determined by the frequency of off-target mutations). Amplicon sequencing was used to determine the editing events within *w* locus in individuals from WhA-D crosses and for the frequency of mutations at off-target sites in G0 WhA-D and CnA-F insects. Sanger sequencing was used to determine the editing events in G0 CnA-F insects and for determining the alleles in mutant insects from G1-G3 generations. Genomic DNA was extracted from wild-type and G0 *cn* and *w* GWSS mutants as described in Supplemental Methods. The methods for PCR amplification of target/off-target regions, Illumina library construction, amplicon or Sanger sequencing of loci are described in Supplemental Methods. Primers are in Supplementary Table S5.

### Pteridine extraction and quantification

Pteridines were extracted using published methods^60^. Two wings (forewings or hindwings) and one head from 1- to 3-day old GWSS adults were dissected and weighted on a precision scale. The issues were grounded in liquid nitrogen with a plastic pestle for 60 s and homogenized in 0.5 mL of 1:1 0.1% aqueous ammonia and chloroform. The samples were centrifuged for 2 min at 10,000*g*. The aqueous phase was collected and the absorbance measured on a NanoPhotometer NP80 (Implen). Emission spectra between 200 and 700 nm were recorded. Five replicates per genotype were used and aqueous ammonia (0.1%) was used as a control. 6-biopterin (Sigma-Aldrich: Munich, Germany) is one of the major pteridines founded in extracts of insect heads^34,35^, and it was used as control. 6-biopterin was diluted in 0.1% aqueous ammonia (1 mg/mL). Serial dilutions from the 6-biopterin stock solution from 0.02 to 0.000121 mg/mL as well as blank samples.

## Supplementary Information

Supplementary Information 1.

Supplementary Information 2.

Supplementary Information 3.

Supplementary Information 4.

Supplementary Information 5.

Supplementary Information 6.

Supplementary Information 7.

Supplementary Information 8.

Supplementary Information 9.

Supplementary Information 10.

## Data Availability

Amplicon library NGS data and Sanger sequencing for G0 individuals are supplied, as is the Sanger sequencing of G1, G2 and G3 individuals. All other data and strains are available upon request to the authors.
