# Supplementary material for: Efficient CRISPR/Cas9-mediated genome modification of the glassy-winged sharpshooter Homalodisca vitripennis (Germar)
Source: Sci Rep. 2022 Apr 19;12:6428. doi: 10.1038/s41598-022-09990-4 (PMC9018754; doi:10.1038/s41598-022-09990-4)
Supplement: Supplementary file 1 — Supplementary Information 1. [file 41598_2022_9990_MOESM1_ESM.pdf]

Supplementary Figure S1

a

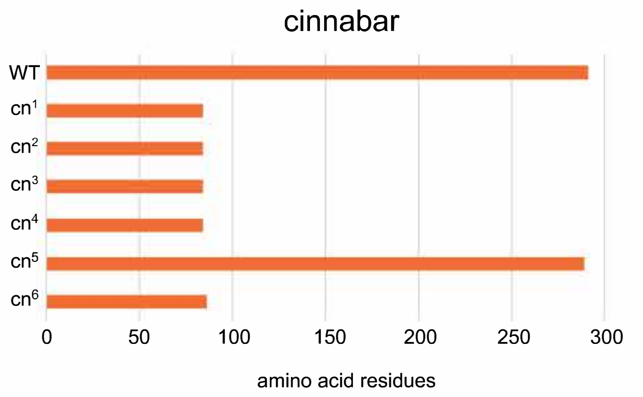

c

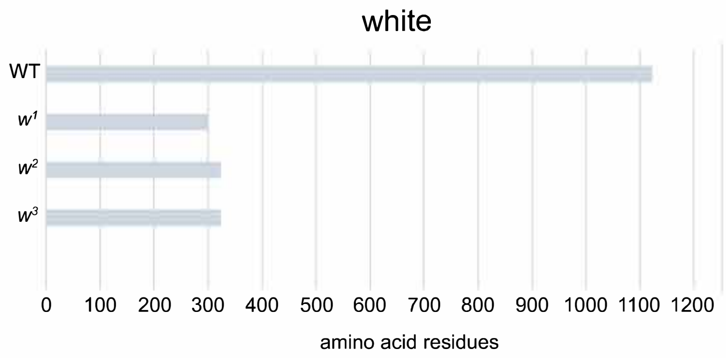

b

Wild-type *cn* deduced protein (291 residues)

MKRPMFDYNQTYIQHGYMELCIPPTQDGQFAMKPNYLHIWPRGSFMMIALPNQDCSWTVTLFMPFSQF**SALDSPVRL**
FNTHFPDSISLIGKEKLVHDYFAASPSNLVSVKCGPYNVGSSAVLIGDAAHAMVPFYGQGMNAGFEDCQLLDRILTESGW
DLQTALPQFSSFRRPDAHAICDLAMYNYVEMRDLVTRQSFLWRKRLDNALYWMFPSLWVPLYNSVTFSDMRYSHCASNKR
WQDKMVSRGLRVACVFLCLTTVYCSVSWWPSLRQTLHPHFTSVFLFSPLLS*

***cn*[1] deduced protein (84 residues)**

MKRPMFDYNQTYIQHGYMELCIPPTQDGQFAMKPNYLHIWPRGSFMMIALPNQDCSWTVTLFMPFSQF**SALDSPVRLLL**P
HPLP*

***cn*[2] deduced protein (84 residues)**

MKRPMFDYNQTYIQHGYMELCIPPTQDGQFAMKPNYLHIWPRGSFMMIALPNQDCSWTVTLFMPFSQF**SALDSPVRQLL**P
HPLP*

***cn*[3] deduced protein (84 residues)**

MKRPMFDYNQTYIQHGYMELCIPPTQDGQFAMKPNYLHIWPRGSFMMIALPNQDCSWTVTLFMPFSQF**SALDSPVRQVI**P
HPLP*

***cn*[4] deduced protein (84 residues)**

MKRPMFDYNQTYIQHGYMELCIPPTQDGQFAMKPNYLHIWPRGSFMMIALPNQDCSWTVTLFMPFSQFS**ALDSPVRLQL**L
PHPLP*

***cn*[5] deduced protein (289 residues)**

MKRPMFDYNQTYIQHGYMELCIPPTQDGQFAMKPNYLHIWPRGSFMMIALPNQDCSWTVTLFMPFSQF**SALDSPVR**NFFH
THFPDSISLIGKEKLVHDYFAASPSNLVSVKCGPYNVGSSAVLIGDAAHAMVPFYGQGMNAGFEDCQLLDRILTESGWDL
QTALPQFSSFRRPDAHAICDLAMYNYVEMRDLVTRQSFLWRKRLDNALYWMFPSLWVPLYNSVTFSDMRYSHCASNKRWQDK
MVSRGLRVACVFLCLTTVYCSVSWWPSLRQTLHPHFTSVFLFSPLLS*

***cn*[6] deduced protein (86 residues)**

MKRPMFDYNQTYIQHGYMELCIPPTQDGQFAMKPNYLHIWPRGSFMMIALPNQDCSWTVTLFMPFSQF**SALDSP**SSTPTS
LTPSVS*

d

Wild-type *w* protein (1161 aa)

MLIREVANKVIEMPCAQNHRNGSQKELSNGLYLELSDTEKKPQVNGYSSGSGGSGSPYSTVSILPKENITYTWSNVNVFT
RLEHRQNRVVNLVHNVVHRDRPQQRKHILKNVTGVAYPGELMALMGSSGAGKTTLLNSLTFRAPKTLAVTGHRAINGVPV
DANALAALSAYVQQDDLFIGTLTVREHLIFQALVRMDRHIPYVQRMIRVEEVINDLMLTRCQDTVIGVPGKIKGISGGEM
KRLSFASEVLTNPPLMFCDEPTSGLDSFMAQNVVTVLKSLAE**KGKTIVCTIHQPSSEVFSMFD**KILLMAEGRVAFLGTPS
EAIDFFKTSVLLLLSMYSMKNKTNKYPCGKCLANVSKCSKAVLCKGSCNQWLHLKCTDLSKEDYERIKKSIIKKWLCSNC
ELADIDEEIEVEDRVLYVELKEELESQELIIKNLSEDLAKANDEIKNVSTYTLNLETLVLKKEESIELLERKVKEISENK
SECKCLNKYKATGPRRSLPAKLSTPVSSIMNKNNRLDSEKNKELPYTVVKNRKQKHATNIIFQNKDLNFISKNQYEVLSV
EEEEEENGPKEQPVQTSSREKKKLLICADSHGRDLAWNLNQTQHTYEAVGFVRPGGRTGQVLDNHNIEEEKLGEEDGLVI
LCGTNDVAINNAQDAIKNIELVLNKITSTVSKIVVVDIPRRYDLVEWSCVNQEVKKTNCALKEMCNKFRDVSLVEVSRAE
RHLHTRHGMHLNRRGKMWLAHQIARVLDSKQEQETYNVSPTLRTGSEESVADAMMTTEISTPTTEDSTTSSGNSPLQTPT
HNRMGAVCPSHYNPADFFIQLLAVVPNAEESCRNMIEMVCDSFATSEIGSKISMLAEVGGGTKVKSAWNWSDPFNNSVSM
YKASWCAQFRAVFWRSWLSVNKEPVLIKVRILQTLMVSLMVGIIYFGQDYDQDGVMNINGALFICISNMTFQNVFAVITV
FCSEMPVFMREHYNGMYRTDVYFLCKTLAEVPIFLVIPVLFTCIMYYMVGLNPDPSRFFQSIVIITLVSNVATSFGYFIS
SVSSSIAVALSIGPPIIIPFLLFGGFFLNVGSVPPYFKWLSHLSWFKYGNEALLINQWDNIKDIACTRSNTTCPSDGHVV
LEQFNFSERNYIWDYIWLVVLIIGFRVVAFLALLYRTSRRR*

***w*[1] deduced protein (299 residues)**

MLIREVANKVIEMPCAQNHRNGSQKELSNGLYLELSDTEKKPQVNGYSSGSGGSGSPYSTVSILPKENITYTWSNVNVFT
RLEHRQNRVVNLVHNVVHRDRPQQRKHILKNVTGVAYPGELMALMGSSGAGKTTLLNSLTFRAPKTLAVTGHRAINGVPV
DANALAALSAYVQQDDLFIGTLTVREHLIFQALVRMDRHIPYVQRMIRVEEVINDLMLTRCQDTVIGVPGKIKGISGGEM
KRLSFASEVLTNPPLMFCDEPTSGLDSFMAQNVVTVLKSLAE**KGKTIVCTIHQPSS**VCI*

***w*[2] deduced protein (323 residues)**

MLIREVANKVIEMPCAQNHRNGSQKELSNGLYLELSDTEKKPQVNGYSSGSGGSGSPYSTVSILPKENITYTWSNVNVFT
RLEHRQNRVVNLVHNVVHRDRPQQRKHILKNVTGVAYPGELMALMGSSGAGKTTLLNSLTFRAPKTLAVTGHRAINGVPV
DANALAALSAYVQQDDLFIGTLTVREHLIFQALVRMDRHIPYVQRMIRVEEVINDLMLTRCQDTVIGVPGKIKGISGGEM
KRLSFASEVLTNPPLMFCDEPTSGLDSFMAQNVVTVLKSLAE**KGKTIVCTI**CAPKTIGILHVRQDLAHGRGSCGIPWNSI
RSH*

***w*[3] deduced protein (324 residues)**

MLIREVANKVIEMPCAQNHRNGSQKELSNGLYLELSDTEKKPQVNGYSSGSGGSGSPYSTVSILPKENITYTWSNVNVFT
RLEHRQNRVVNLVHNVVHRDRPQQRKHILKNVTGVAYPGELMALMGSSGAGKTTLLNSLTFRAPKTLAVTGHRAINGVPV
DANALAALSAYVQQDDLFIGTLTVREHLIFQALVRMDRHIPYVQRMIRVEEVINDLMLTRCQDTVIGVPGKIKGISGGEM
KRLSFASEVLTNPPLMFCDEPTSGLDSFMAQNVVTVLKSLAE**KGKTIVCTIHQPSS**VISILHVRQDLAHGRGSCGIPWNS
IRSH*

FAD-binding domain Transmembrane domain ATPase domain ABC domain
