# Supplementary figures and images for: Efficient CRISPR/Cas9-mediated genome modification of the glassy-winged sharpshooter Homalodisca vitripennis (Germar)
Source: Sci Rep. 2022 Apr 19;12:6428. doi: 10.1038/s41598-022-09990-4 (PMC9018754; doi:10.1038/s41598-022-09990-4)

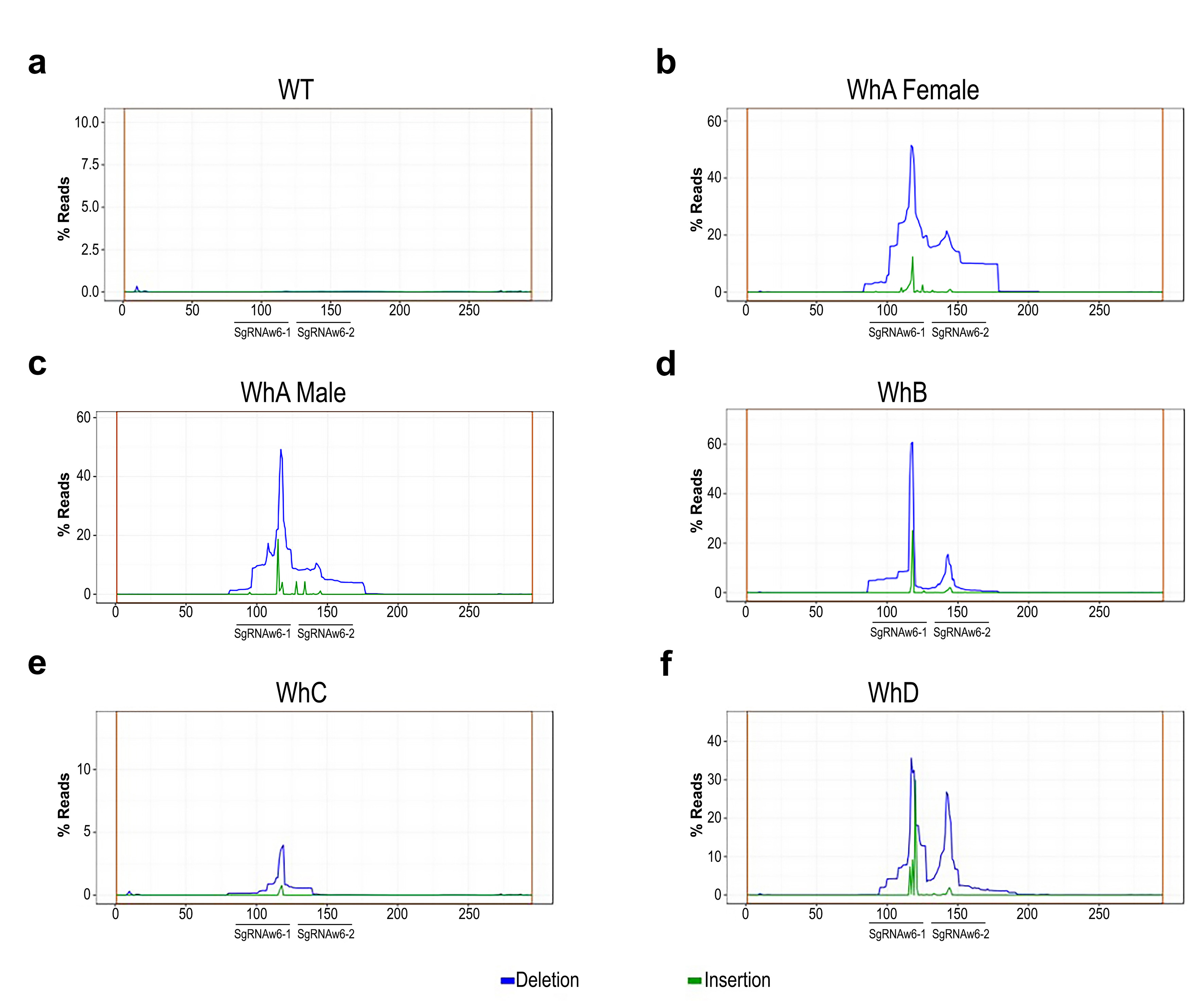

Supplement: Supplementary file 2 — Supplementary Information 2. [file 41598_2022_9990_MOESM2_ESM.jpg]

Supplementary Figure S3

M CnA CnB CnC CnD CnE CnA CnA CnA Cn1 Cn2 WT N

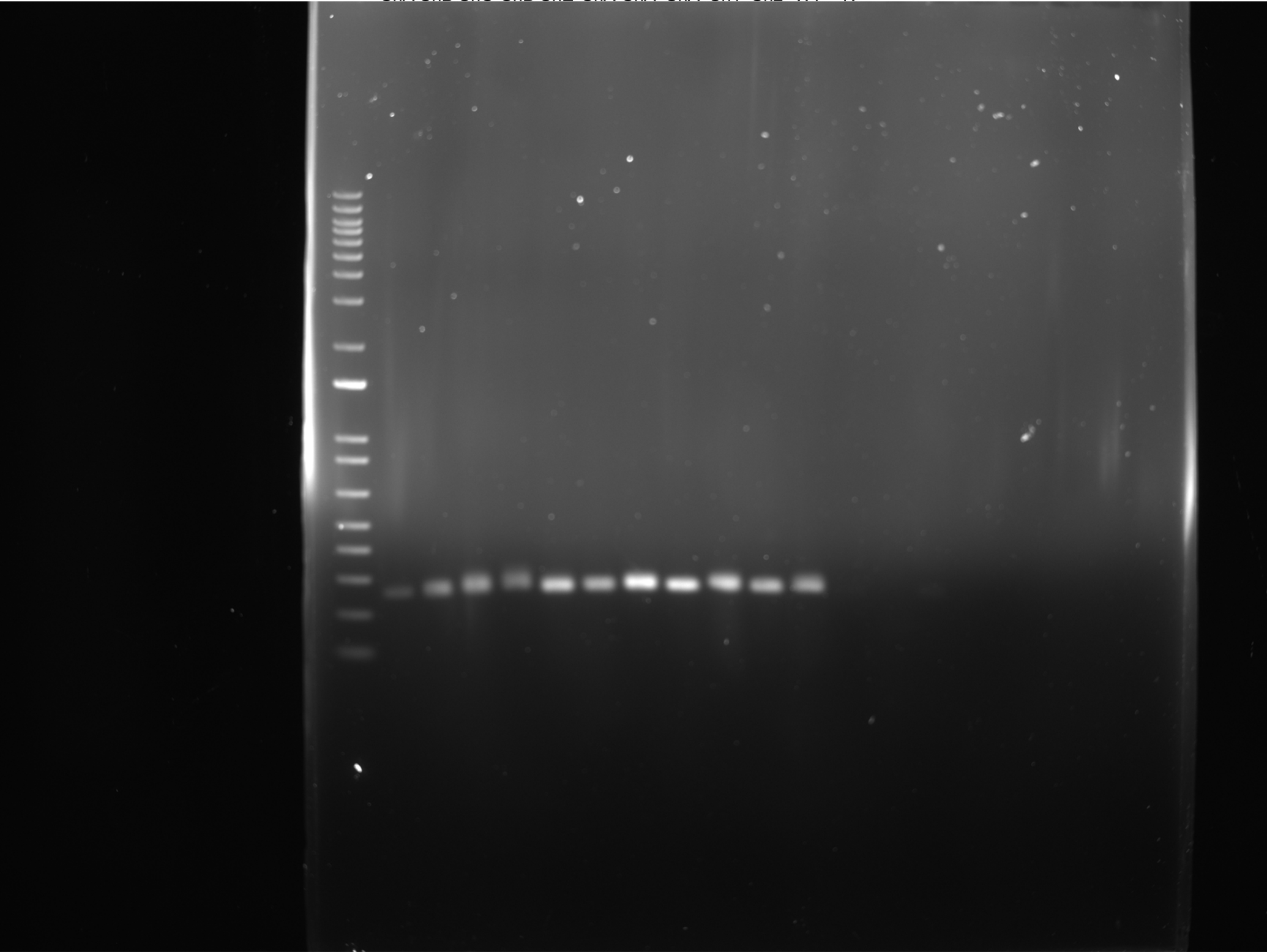

Supplement: Supplementary file 3 — Supplementary Information 3. [file 41598_2022_9990_MOESM3_ESM.pdf]
