# Supplementary material for: Efficient CRISPR/Cas9-mediated genome modification of the glassy-winged sharpshooter Homalodisca vitripennis (Germar)
Source: Sci Rep. 2022 Apr 19;12:6428. doi: 10.1038/s41598-022-09990-4 (PMC9018754; doi:10.1038/s41598-022-09990-4)

Supplementary Figure S4

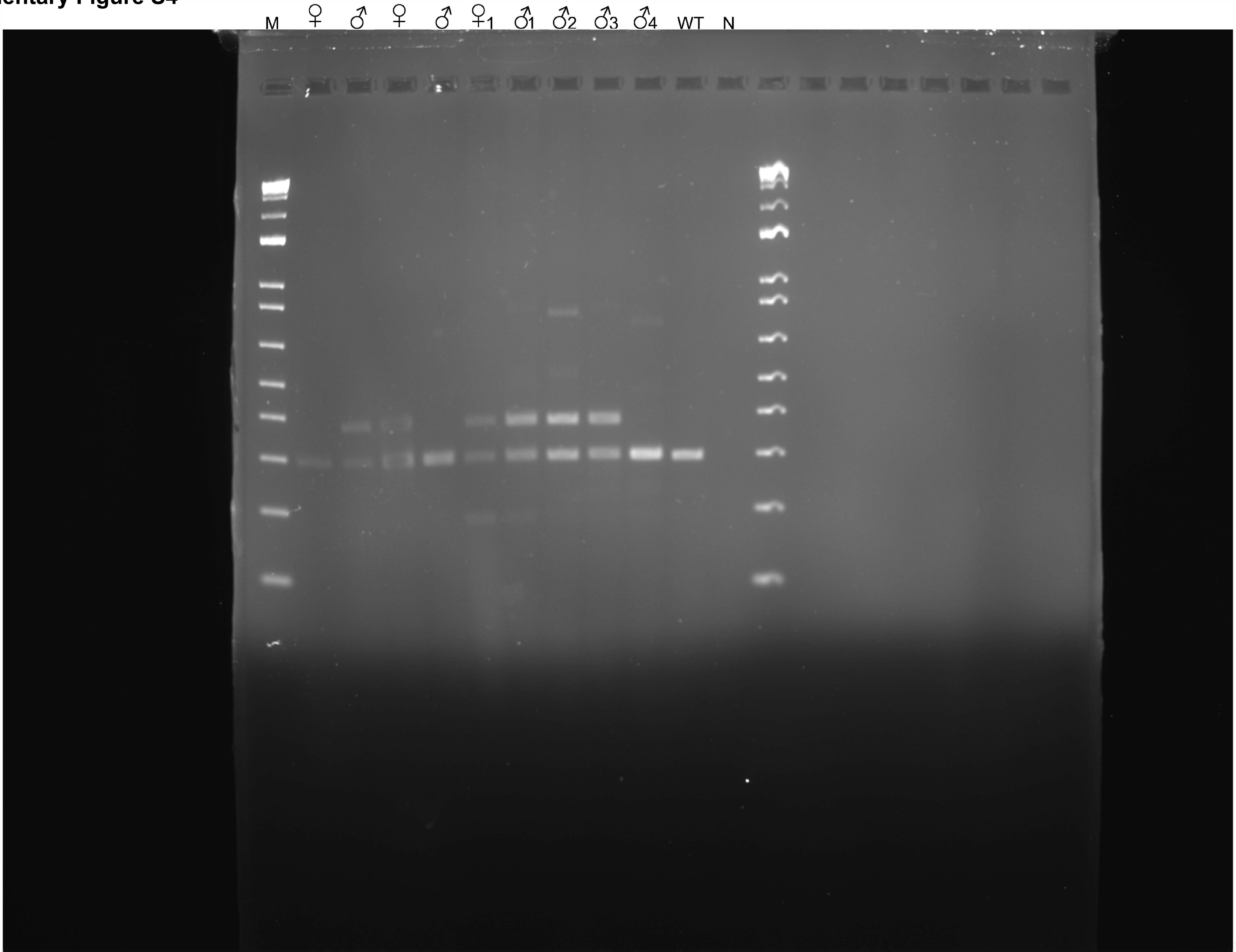

Supplement: Supplementary file 4 — Supplementary Information 4. [file 41598_2022_9990_MOESM4_ESM.pdf]
