# Supplementary material for: Efficient CRISPR/Cas9-mediated genome modification of the glassy-winged sharpshooter Homalodisca vitripennis (Germar)
Source: Sci Rep. 2022 Apr 19;12:6428. doi: 10.1038/s41598-022-09990-4 (PMC9018754; doi:10.1038/s41598-022-09990-4)
Supplement: Supplementary file 5 — Supplementary Information 5. [file 41598_2022_9990_MOESM5_ESM.pdf]

Supplementary Information

Supplementary Methods

CRISPR-Cas9 reagents

Synthesis of *white* sgRNAs: Two *w* sgRNAs (sgRNAw6-1 and sgRNAw6-2) were initially synthesized *in vitro* (Supplemental Table S5). *In vitro* transcription templates to produce the *white* sgRNAs were synthesized using two partially overlapping oligonucleotides (Tail primer and sgRNAw6-1 or sgRNAw6-2) (Supplementary Table S5). The oligos were annealed and used in a "template-free" PCR to obtain a linear double-stranded DNA template. The reaction was performed 20 µl 5× Q5 reaction buffer, 2 µl dNTP Mix (10 µM), 2.5 µl of each primer (10 µM) and 1 µl Q5 high-fidelity polymerase (2 U) (New England Biolabs, NEB, MA, US) in a total volume of 100 µl. PCR reactions were run in a T100 Thermal Cycler (BioRad, California, USA) using the conditions: 98ºC for 30 s; 35 cycles of 98ºC for 10 s, 60ºC for 30 s; and 72ºC for 10 min. The sgRNA templates were purified using Monarch DNA Cleanup kit (NEB) accordingly to the manufacturer's instructions. Using 500 ng of purified DNA template, *in vitro* transcription reactions were performed overnight at 37 °C using a Megascript T7 transcription kit according to the manufacturer's instructions (Thermofisher, California, USA). sgRNAs were purified using MEGAclear Transcription Clean-Up Kit (Thermofisher) following the manufacturer's instructions.

cinnabar and *white* sgRNAs were also purchased from Synthego (CRISPRevolution sgRNA EZ Kit, California, USA) (Supplemental Table S5). The sgRNAs were resuspended in nuclease-free water to a final concentration of 100 µM following manufacturer's protocols. All injections targeting the c*n* locus were performed using the sgRNAcn4-1 (Synthego). All injections targeting the *w* locus were performed with sgRNAs prepared in our laboratory with the exception of the injections performed in the embryos shown in Figure 1e in which the Synthego-synthesized *w* sgRNAs were used.

Injection mixes: Injection mixes were prepared on ice with 300 ng/µl Cas9 protein (PNA Bio Inc) and sgRNA (300 ng/µl or 150 ng/µl) in a volume of 50 µl. All mixes were incubated at 37°C for 5 min to pre-assemble the Cas9 and sgRNAs. CRISPR mixes were aliquoted (2 µl/tube) and stored at -80°C until use. Injection mixes were thawed on ice prior to use and used once.

Needle preparation and microinjection

Quartz capillaries with filament (QF100-70-10, 10-cm long, 0.7-mm internal diameter and 1.0-mm external diameter, Sutter Instruments, Novato, CA USA) were siliconized as follows: 10 mL of Sigmacote (Sigma) was placed into a 50 mL glass beaker and allowed to enter each by capillary action. These were then inverted end to end 6X to ensure that the entire length of each capillary was coated. The capillaries were drained and blot dried on a Kimwipe and then the remaining solution blown out of each using a WPI Picopump (World Precision Instruments, Sarasota, FL USA). The siliconized capillaries were then oven dried at 200°C overnight. These were pulled into needles using a P-2000 Micropipette Laser puller with a glass stop (GS-1) placed in one loading clamp to ensure needles of equal length (Sutter Instruments, Novato, CA USA). The parameters used were: Heat, 100; Filament, 4; Velocity, 45; Delay, 135; Pull, 160. Needles were beveled to 30° using a BV-10 beveler (Sutter Instruments, Novato, CA USA), an ultrafine grinding plate in 1% PhotoFlow (Kodak, Rochester, NY USA) using air pressure to determine the level of needle opening. The injection station consisted of a stereoscope (Olympus SZH10 Research, Tokyo, Japan), TrioMPC-100 manipulator (Sutter Instruments, Novato, Ca USA) and Digital Microinjector (XenoWorks, Sutter Instruments, Novato, CA USA). The angle of attack of the needle was 30-40° with the D axis control of the manipulator used for needle penetration of each egg to minimize leakage.

Screening and image acquisition

The developing embryos and emerged nymphs were screened for altered eye color using a stereomicroscope. Wild-type GWSS embryos and first-instar nymphs have red-brown eye color[1], while adults have dark-brown eyes with yellow striations and wings with red pigments in some interveinal spaces (cells) and veins[2]. Eye color is discernable in embryos 5 d after egg deposition. GWSS nymphs with an edited *white* gene have white or mosaic eyes, while nymphs with an edited *cinnabar* gene have red-orange or mosaic eyes. The % embryos and generation 0 (G0) nymphs with mutant eye color were determined. G0 mutant nymphs were transferred to cages with six sunflower plants to complete their developmental cycle[3] (see Methods in manuscript for rearing details).

Photographs of developing embryos and nymphs, as well as the eyes, wings, and bodies of adult wild-type, G0-G4 *w* and G0-G3 *cn* GWSS were taken with a Toup Tek Industrial Digital Camera 20MP 1, Sony Exmor CMOS sensor, and 4K UHD Multi-output HDMI Camera coupled

to a LEICA M165 FC stereoscope using Toup View camera program. Bright-field images were taken using auto settings for exposures and white balance. Adobe Photoshop 2020 was used to apply moderate changes to image brightness and contrast. Changes were applied equally across the entire image and for all images.

Confirmation of mutations

DNA extraction: GWSS genomic DNA was obtained from a distal portion of the adult GWSS abdomen. DNA extraction was performed according to the manufacturer's instructions using DNeasy Blood & Tissue (QIAGEN, Düsseldorf, Germany). Genomic DNA was quantified using a NanoDrop 2000c spectrophotometer (NanoDrop Technologies, Delaware, USA).

Amplification of *white* and *cinnabar* loci: The genotypes of G0-G3 *w* and G0-G2 *cn* mutants were determined. Forward and reverse primers for *w* (exon 6) and *cn* (exon 4) (Supplementary Table S5) were used to amplify the target regions generating 294-bp and 264-bp fragments, respectively. The PCR reaction was conducted using 100 ng of GWSS genomic DNA, 10 µl 5x Q5 reaction buffer, 1 µl dNTP Mix (10 µM), 1.25 µl of each gene-specific primer (10 µM), and 0.5 µl Q5 high-fidelity polymerase (1 U, NEB) in a total volume of 50 µl. The reaction was performed in a T100 Thermal Cycler (98°C, 30s; 35 cycles of 98°C for 10 s, 64°C for 20 s, and 72°C for 30 s; and 72°C for 2 min). The amplicons were fractionated by 0.8-2% agarose electrophoresis and visualized after ethidium bromide staining. The PCR products were purified using QIAquick PCR Purification Kit (Qiagen) as recommended in the manufacturer's protocol.

For the G0 *white* GWSS, amplicon sequencing was performed. A second PCR cycle was performed to add Illumina adaptors (Supplemental Table S5) to the amplicons. This PCR was carried out using 100-200 ng of purified *w* amplicon, 20 µl 5 × Q5 reaction buffer, 2 µl dNTP Mix (10 µM), 2.5 µl of E1_Ez-primers (10 µM) and 1 µl Q5 high-fidelity polymerase (2 U) in a total volume of 100 µl. The PCR protocol was 98°C for 30 s; 14 cycles of 98°C for 10 s, 58°C for 15 s, and 72ºC for 30s; 43 cycles of 98°C for 10 s, 72°C for 40 s; and 72°C for 5 min. Integrity and quantity of amplicons were verified by 0.8% agar electrophoresis, NanoDrop 2000c (Thermo Scientific) and Qubit 2.0 analysis (Thermofisher). PCR products were purified using QIAquick PCR Purification Kit (Qiagen) and 500 ng of PCR product were sequenced on an Illumina platform (Genewiz, CA, USA).

The changes in the *cn* gene in the G0 CnA-F insects and G1 and G0 CnA was determined by Sanger sequencing of the QIAquick-purified PCR products. In addition, genotypes of WhA G1-

G3 adults was determined in a similar manner. QIAquick-purified *w* and *cn* PCR products were cloned into the pJet vector (Thermofisher) according to the manufacturer's instructions and transformed into *Escherchia coli* JM109 chemically competent cells according to standard procedures (Zymo research, California, USA). Ampicillin-resistant clones were identified and colony PCR was performed using the AccuPower PCR PreMix (Bioneer, California, USA), 1 µl liquid culture and 2 µl of pJet primers (10 µM) in a final volume of 20 µl. The PCR protocol was: 96°C for 6 min, 25 cycles of 94°C for 20 s, 52°C for 20 s, 72°C for 2 min; and 72°C for 7 min. The PCR products were purified using QIAquick PCR Purification Kit. Integrity and quantity of amplicons were verified by 0.8% electrophoresis gel and NanoDrop 2000c (Thermo Scientific) analysis, respectively. The purified PCR fragments (300-500 ng) were sequenced by Retrogen Inc (California, USA) using the pJet vector primers. The amplicon sequences from G0, G1, G2, and G3 insects were analyzed *via* alignments to wild-type GWSS sequences using Cas-analyzer tool[4] and the MUSCLE alignment tool[5].

Cleavage efficiency of sgRNA *in vitro*: The cleavage efficiency of *white* and *cinnabar* sgRNAs were evaluated using the Guide-it sgRNA Screening System kit (Takara Bio, Shiga, Japan). sgRNA (50 ng) and Guide-it Cas9 (250 ng) in a final volume of 1.5 µl were assembled at 37°C for 5 minutes. At this time, 30-100 ng of the WT PCR amplicon was added to the tube, followed by 1 µl of 15 x Cas9 buffer, 1 µl of 15 x BSA and 6.5 µl of RNase-free water (total volume of 20 µl). The reaction was incubated at 37°C for one h, followed by 80°C for five min. The digested fragments were fractionated in a 1.5% agarose gel.

Analysis and sequencing of off-target loci

We selected for off-target sequences that contained 3-4 mismatches distal to the seed regions of the three sgRNA target sites with bulge sizes from 0-2 using Cas-OFFinder [6] (Supplementary Table S4b). Genomic DNAs from WhA-D G0 and CnA-F G0 adults prepared as described above were used to characterize the frequency of insertions and deletions at off-target loci. The methods for PCR amplification of off-target regions, Illumina library construction, and amplicon sequencing of loci were performed as described for the *w* and *cn* loci in G0 insects. Off-target locus-specific primers and their T_ms are provided in Supplemental Table S5b.

References

1. Al-Wahaibi, A.K. & Morse, J.G. Egg morphology and stages of embryonic development of the glassy-winged sharpshooter (Hemiptera: Cicadellidae). *Ann. Entomol. Soc. Am.* **102**, 241-248 (2009).
2. Timmons, C., Hassell, A., Lauziere, I. & Bextine, B. Age determination of the glassy-winged sharpshooter, *Homalodisca vitripennis*, using wing pigmentation. *J Insect Sci* **11**, 78 available online: insectscience.org/11.78 (2011).
3. Sisterson, M.S. Egg load dynamics of Homalodisca vitripennis. *Environ Entomol* **37**, 1200-1207 (2008).
4. Park, J., Lim, K., Kim, J.S. & Bae, S. Cas-analyzer: an online tool for assessing genome editing results using NGS data. *Bioinformatics* **33**, 286-288 (2017).
5. Edgar, R.C. MUSCLE: multiple sequence alignment with high accuracy and high throughput. *Nucleic Acids Res.* **32**, 1792-1797 (2004).
6. Bae, S., Park, J. & Kim, J.S. Cas-OFFinder: a fast and versatile algorithm that searches for potential off-target sites of Cas9 RNA-guided endonucleases. *Bioinformatics* **30**, 1473-1475 (2014).

Supplementary Table Titles

The supplementary tables are in separate attachments

Supplementary Table 1. Nymph emergence from GWSS eggs five to ten days post-injection. GWSS embryos were injected with water *in situ* and allowed to develop for 10 d. Number of embryos with developing eyes is an indicator of embryo survival. Nymph emergence was monitored from 5 to 10 dpi; synchrony in nymph emergence was observed.

Supplementary Table 2a. Sanger sequencing of the *cn* target region from the GWSS G0 mutant CnA.

Supplementary Table 2b. Sanger sequencing of the *cn* target region from the GWSS G0 mutant CnB.

Supplementary Table 2c. Sanger sequencing of the *cn* target region from the GWSS G0 mutant CnC.

Supplementary Table 2d. Sanger sequencing of the *cn* target region from the GWSS G0 mutant CnD.

Supplementary Table 2e. Sanger sequencing of the *cn* target region from the GWSS G0 mutant CnE.

Supplementary Table 2f. Sanger sequencing of the *cn* target region from the GWSS G0 mutant CnF

Supplementary Table 3a. Total reads of recovered *w* mutations from G0 adults.

Supplementary Table 3b. Frequencies of recovered *w* mutations from G0 adults.

Supplementary Table 3c. Numbers and frequencies of unique *w* alleles from G0 adults based on mutation type.

Supplementary Table 3d. Sequences of mutations recovered from wild-type female.

Supplementary Table 3e. Sequences of mutations recovered from WhA G0 female.

Supplementary Table 3f. Sequences of mutations recovered from WhA G0 male.

Supplementary Table 3g. Sequences of mutations recovered from WhB G0 female.

Supplementary Table 3h. Sequences of mutations recovered from WhC G0 female.

Supplementary Table 3i. Sequences of mutations recovered from WhD G0 female.

Supplementary Table 4a. Summary of the % indels in G0 females at putative off-target sites from the w and cn experiments.

Supplementary Table 4b. Off-target gene ID numbers**.**

Supplementary Table 4c. Frequencies and real values of off-target effects of sgRNAw6-1 across 5 target sites in WhA, WhB, WhC and WhD G0 females**.**

Supplementary Table 4d. Frequencies and real values of off-target effects of sgRNAw6-2 across 4 target sites in WhA, WhB, WhC and WhD G0 females**.**

Supplementary Table 4e. Frequencies and real values of off-target effects of sgRNAcn4-1 across 3-4 target sites in CnA, CnB, CnC, CnD, and CnE G0 females**.**

Supplementary Table 5. Primer and sgRNA sequences used in this study.

Supplementary Figure Legends

Supplementary Figure S1. Conceptual translation of wild-type and mutant w and cn proteins.

(a) The length of the conceptual white protein sequences of the wild-type, w^1, w^2 and w^3 alleles. (**b)** Amino acids sequence of the wild-type and three mutant conceptual *w* proteins with the AAA (brown), ABC (teal), and transmembrane (maroon) domains shown. **(c)** The length of the conceptual cinnabar protein sequences of wild-type and five *cn* alleles (cn^1-cn^5). **(d)** Amino acids sequence of the wild-type *cn* and predicted cn^1-cn^5 allele proteins with the FAD-binding domain (blue), and two transmembrane (maroon) domains shown. Sequences in the target region in w and cn are shown in red.

Supplementary Figure S2. The frequency of insertion/deletion events in wild-type and four G0 *w* mutants.

The *w* target region was PCR amplified from G0 GWSS adults and prepared for amplicon sequencing. The Genewiz report provided documentation of the number of insertions, deletions and substitutions (not shown) in the *w* target region for wild-type and six *w* G0 mutant GWSS. **(a)** wild-type, **(b)** female parent of the WhA line, **(c)** male parent of the WhA line, **(d-f)** female parents of the WhB, WhC and WhD lines, respectively. Frequency of insertions (green) and deletions (blue) are shown. Indels in GO GWSS were at low levels in wild-type and WhC adults. Primary data can be found in Supplementary Tables 3d-i.

Supplementary Figure S3.

The complete photograph of the gel shown in Figure 4d. Lane N is the no DNA template control.

Supplementary Figure S4

The complete photograph of the gel shown in Figure 6d. Lane N is the no DNA template contro
