# Supplementary material for: Efficient CRISPR/Cas9-mediated genome modification of the glassy-winged sharpshooter Homalodisca vitripennis (Germar)
Source: Sci Rep. 2022 Apr 19;12:6428. doi: 10.1038/s41598-022-09990-4 (PMC9018754; doi:10.1038/s41598-022-09990-4)
Supplement: Supplementary file 6 — Supplementary Information 6. [file 41598_2022_9990_MOESM6_ESM.docx]

| **No. of egg masses** | **No. of eggs** | **No. with eye discs** | **Number of emerged nymphs**  **(days post-injection, dpi)** | | | | | | **No. of nymphs emerged** | **% hatch** |
| --- | --- | --- | --- | --- | --- | --- | --- | --- | --- | --- |
|  |  |  | **5 dpi** | **6 dpi** | **7 dpi** | **8 dpi** | **9 dpi** | **10 dpi** |  |  |
| 11 | 112 | 77 | 0 | 1 | 65 | 5 | 1 | 0 | 72 | 64.3 |

**Table S1.** Nymph emergence from GWSS eggs five to ten days post-injection. GWSS embryos were injected with water *in situ* and allowed to develop for 10 d. Number of embryos with developing eye discs is an indicator of embryo survival. Nymph emergence was monitored from 5 to 10 dpi; synchrony in nymph emergence was observed.
